# Heteroleptic Copper(II) Complexes Containing 2′-Hydroxy-4-(Dimethylamino)Chalcone Show Strong Antiproliferative Activity

**DOI:** 10.3390/pharmaceutics15020307

**Published:** 2023-01-17

**Authors:** Zdeněk Trávníček, Tomáš Malina, Ján Vančo, Marek Šebela, Zdeněk Dvořák

**Affiliations:** 1 Regional Center of Advanced Technologies and Materials, Czech Advanced Technology and Research Institute (CATRIN), Palacký University, Šlechtitelů 241/27, CZ-783 71 Olomouc, Czech Republic; 2 Department of Biochemistry, Faculty of Science, Palacký University, Šlechtitelů 27, CZ-783 71 Olomouc, Czech Republic; 3 Department of Cell Biology and Genetics, Faculty of Science, Palacký University, Šlechtitelů 27, CZ-783 71 Olomouc, Czech Republic

**Keywords:** copper(II) complexes, chalcone, in vitro cytotoxicity, antiproliferative activity, cell cycle, cell death, mitochondrial membrane potential, reactive oxygen species

## Abstract

A series of six heteroleptic copper(II) complexes with 2′-hydroxy-4-(dimethylamino)chalcone (HL) with the composition [Cu(N-N)(L)]NO_3_ (**1**–**6**), where N-N stands for dmbpy = 5,5′-dimethyl-2,2′-bipyridine (**1**), bphen = 4,7-diphenyl-1,10-phenanthroline (**2**), dbbpy = 4,4′-di-*tert*-butyl-2,2′-bipyridine (**3**), nphen = 5-nitro-1,10-phenanthroline (**4**), bpy = 2,2′-bipyridine, (**5**), and dpa = 2,2′-dipyridylamine (**6**), was prepared and thoroughly characterized. The in vitro cytotoxicity screening on eight human cancer cell lines identified complex **2**, containing the bulkiest *N*-donor ligands (bphen) as highly cytotoxic against cancer cells, with IC_50_ values ranking from 1.0 to 2.3 μM, with good selectivity and low toxicity against healthy human fetal lung fibroblasts MRC-5. The cell-based assays, involving the most effective complex **2** in A2780 cancer cells, revealed its strong pro-apoptotic effects based on the effective activation of caspases 3/7, ROS overproduction, and autophagy in the A2780 cells while not impeding the cell cycle and mitochondrial membrane functions. The cellular uptake studies in A2780 and 22Rv1 cells uncovered no intracellular transport of the cationic complex **2**, supporting the hypothesis that the in vitro anticancer effects of complex **2** are based on the combined extrinsic activation of apoptosis and autophagy induction.

## 1. Introduction

Chalcones (1,3-diphenyl-2-en-1-ones) represent a group of naturally occurring compounds that serve as synthetic precursors for different classes of flavonoids. They are present in a variety of eatable plants and they show a wide range of biological activities such as antioxidant, anticancer, antibacterial, anti-inflammatory, antimalarial, antifungal, and antiviral. For these reasons they have also been used for a long time in traditional medical practice and the biosynthesis and biological features of chalcones have been reviewed in the literature (e.g., [1]). The above-mentioned properties of free chalcones together with their chemical compositions and structures have inspired coordination chemists to synthesize and characterize diverse transition metal complexes with the aim to study not only their biological features, mainly anticancer, antibacterial, antiviral, antioxidant, DNA-binding and cleavage activities, but also to try to find possible applications of such compounds in pharmacotherapy. The structural and biological features of metal complexes have been reviewed in the literature and the biological potential of these agents has been demonstrated [2]. Recent studies support the significance of this research of chalcone-based complexes with transition metals: X. Huang et al. reported a series of Pt(IV) complexes containing chalcone analogues showing excellent in vitro anticancer activities against a panel of human cancer cell lines, with IC_50_ values at submicromolar concentrations [3], and a series of Pt(IV)-chalcone derivatives showing significant-to-moderate in vitro cytotoxicity on selected human cancer cells [4]. D. Dkhar et al. published a paper reporting Ru, Rh, and Ir half-sandwich complexes bearing pyridyl chalcone derivatives that demonstrate potent antibacterial and anticancer activities [5]. R. Kaushal et al. reported a series of vanadyl complexes with chalcone derivatives revealing remarkable antioxidant and antidiabetic activities [6,7]. J. Johnson et al. reported Co, Ni, Cu, and Zn complexes containing a thiazole-based chalcone ligand showing good antimicrobial activity.

**Scheme 1:**
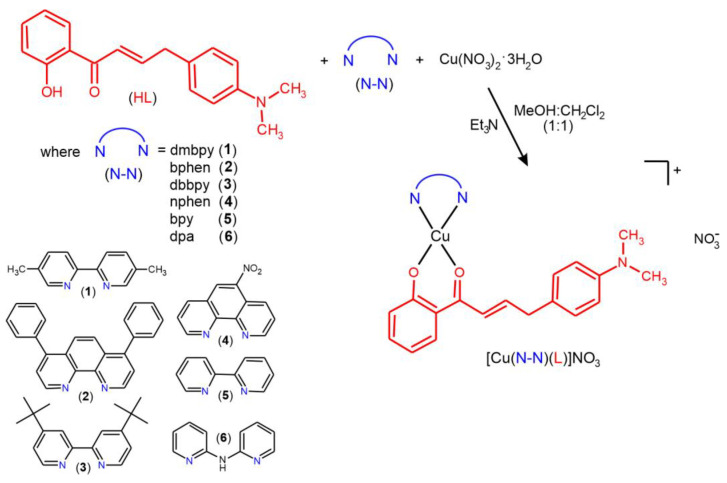
Schematic pathway leading to the preparation of complexes [Cu(N-N)(L)]NO_3_ (**1**–**6**) including the structural formulas of 2′-hydroxy-4-(dimethylamino)chalcone (HL) used as a bidentate *O*-donor ligand together with the group of bidentate *N*-donor heterocyclic diimine ligands.

In our previous study [8], we focused our efforts on the synthesis and characterization of heteroleptic copper(II) complexes with 2′-hydroxychlacone-derived ligands that revealed high in vitro cytotoxicity against a panel of 10 human cancer cell lines, with IC_50_ values ranging from 1.1 μM to 32.6 μM. One of the most anticancer active complexes, having the composition of [Cu(phen)(L)]NO_3_ in solution, where phen = 1,10-phenanthroline and HL = 2′-hydroxy-4-(dimethylamino)chalcone (see Scheme 1), was chosen as a model compound for the present study in which the latter chalcone-ligand was left in the structure of the Cu(II) complexes, but the phen ligand was exchanged for another one from a group of bidentate *N*-donor heterocyclic diimine ligands (see Scheme 1). In this work, we tried to find whether the exchange of the *N*-donor ligand for another one has an impact on the resulting biological features of the complexes.

## 2. Materials and Methods

All of the starting chemicals and solvents were supplied from Sigma-Aldrich Co. (St. Louis, MO, USA) and Merck Co. (Rahway, NJ, USA) and were used as received. The copper(II) complexes (**1**–**6**) were characterized using different techniques as follows: elemental CHN analysis was performed on a Flash Smart CHN analyzer (Thermo Scientific, Waltham, MA, USA). The electronic spectra were recorded on a Perkin-Elmer Lamda 40 spectrometer (Perkin Elmer, Waltham, MA, USA). The conductivity data of the complexes in 10^−3^ M MeCN and MeNO_2_ solutions were obtained at 25 °C using a Cond 340i/SET conductometer (WTW, Rye Brook, NY, USA). FTIR spectra were measured using a Nexus 670 FT-IR (Thermo Nicolet, Waltham, MA, USA) using an ATR technique. Electrospray ionization mass spectra (ESI + MS) were recorded on an LCQ Fleet Ion Trap mass spectrometer (Thermo Scientific, Waltham, MA, USA). Magnetic susceptibility data was measured using an MSB-AUTO device (Sherwood Scientific, Cambridge, UK). Hg[Co(SCN)_4_] was used as a calibrant and diamagnetic corrections were made with Pascal’s constants.

### 2.1. Synthesis and Characterization of the Compounds

2′-Hydroxy-4-(*N*,*N*-dimethylamino)chalcone (HL) was prepared and characterized by the method described in the literature [8]. The copper(II) complexes (**1**–**6**) were prepared by the following general method: the corresponding aromatic diamine ligand (0.5 mmol) was added to a mixture of 0.5 mmol (134 mg) of the HL ligand in MeOH:CH_2_Cl_2_ (1:1, 20 mL) during stirring at room temperature. Afterward, 0.5 mmol (70 μL) of Et_3_N was added drop-wise, which was followed by adding 120 mg (0.5 mmol) of Cu(NO_3_)_2_.3H_2_O in 5 mL of MeOH. The reaction mixture was stirred under reflux for 30 min, subsequently filtered, and left to stand to evaporate at laboratory temperature. The solid phase, which formed after a few days, was filtered off, washed by a cold MeOH (2 × 5 mL), and dried in a vacuum exicator over solid NaOH.

[Cu(dmbpy)(L)]NO_3_ (**1**). Yield: 52%. Anal. calcd. for C_29_H_28_N_4_O_5_Cu (M_r_ = 576.10): C, 60.46; H, 4.90; N, 9.73%. Found: C, 60.35; H, 5.08; N, 9.46%. ESI + MS (MeOH, *m*/*z*): 513.13 (calc. 513.15) [Cu(dmbpy)(L)]^+^, 247.05 (calc. 247.03) [Cu^I^(dmbpy)]^+^. Λ_m_ (MeCN/MeNO_2_, 25 °C, S cm^2^ mol^−1^): 132/76 (electrolyte 1:1). IR peaks (ν_ATR_/cm^−1^): 3036 w, 2900 w, 1596 vs., 1532 s, 1483 vs., 1438 s, 1364 s, 1335 s, 1309 vs., 1254 m, 1181 s, 1152 vs., 1034 s, 983 s, 941 m, 841 m, 814 m, 753 m, 722 m, 659 m, 572 m, 506 m, 426 m, with intensities labelled as: w= weak, m = middle, s = strong, vs. = very strong. UV–Vis (MeCN/MeNO_2_/nujol): λ_max_ (nm): 258, 270, 312, 446/444/262, 316, 424, 542. *µ*_eff_/*µ*_B_ (295 K): 1.85.

[Cu(bphen)(L)]NO_3_ (**2**). Yield: 60%. Anal. calcd. for C_41_H_32_N_4_O_5_Cu (M_r_ = 724.26): C, 67.99; H, 4.45; N, 7.74%. Found: C, 67.84; H, 4.66; N, 7.46%. ESI + MS (MeOH, *m*/*z*): 661.32 (calc. 661.18) [Cu(bphen)(L)]^+^. Λ_m_ (MeCN/MeNO_2_, 25 °C, S cm^2^ mol^−1^): 133/77 (electrolyte 1:1). IR peaks (ν_ATR_/cm^−1^): 3351 m, 3090 w, 3053 w, 2904 m, 2859 m, 2808 m, 1593 vs., 1524 s, 1485 vs., 1442 m, 1406 m, 1362 s, 1305 s, 1230 w, 1153 s, 1030 s, 979 m, 943 m, 857 m, 822 m, 767 m, 729 w, 705 m, 575 m. UV–Vis (MeCN/MeNO_2_/nujol): λ_max_ (nm): 286, 320, 444/450/280, 328, 442, 557. *µ*_eff_/*µ*_B_ (295 K): 1.81.

[Cu(dbbpy)(L)]NO_3_ (**3**). Yield: 67%. Anal. calcd. for C_35_H_40_N_4_O_5_Cu (M_r_ = 660.26): C, 63.67; H, 6.11; N, 8.49%. Found: C, 63.39; H, 6.34; N, 8.25%. ESI + MS (MeOH, *m*/*z*): 597.33 (calc. 597.24) [Cu(dbbpy)(L)]^+^. Λ_m_ (MeCN/MeNO_2_, 25 °C, S cm^2^ mol^−1^): 141/77 (electrolyte 1:1). IR peaks (ν_ATR_/cm^−1^): 3382 w, 3057 w, 2958 w, 2902 w, 2868 w, 1594 vs., 1527 s, 1484 vs., 1440 s, 1358 s, 1325 s, 1303 s, 1250 m, 1175 w, 1147 s, 1028 s, 969 m, 940 m, 851 m, 817 m, 750 m, 718 w, 653 w, 602 w, 573 w. UV-Vis (MeCN/MeNO_2_/nujol): λ_max_ (nm): 294, 308, 450/440/252, 310, 420, 506. *µ*_eff_/*µ*_B_ (295 K): 1.74.

[Cu(nphen)(L)]NO_3_ (**4**). Yield: 58%. Anal. calcd. for C_29_H_23_N_5_O_7_Cu (M_r_ = 617.07): C, 56.46; H, 3.76; N, 11.35%. Found: C, 56.13; H, 3.92; N, 11.12%. ESI + MS (MeOH, *m*/*z*): 661.02 (calc. 661.11) [Cu^I^_2_(L)_2_ + 3H]^+^, 554.10 (calc. 554.10) [Cu(nphen)(L)]^+^, 287.97 (calc. 287.98) [Cu^I^(nphen)]^+^. Λ_m_ (MeCN/MeNO_2_, 25 °C, S cm^2^ mol^−1^): 123/75 (electrolyte 1:1). IR peaks (ν_ATR_/cm^−1^): 3088 w, 3058 m, 2916 w, 2861 w, 2813 w, 1592 s, 1518 s, 1493 vs., 1435 m, 1363 s, 1321 s, 1182 s, 1151 s, 1116 m, 1026 m, 976 m, 940 w, 823 m, 769 m, 716 m, 653 m, 572 m, 520 w. UV-Vis (MeCN/MeNO_2_/nujol): λ_max_ (nm): 276, 314, 444/440/282, 320, 426, 540. *µ*_eff_/*µ*_B_ (295 K): 1.81.

[Cu(bpy)(L)]NO_3_ (**5**). Yield: 73%. Anal. calcd. for C_27_H_24_N_4_O_5_Cu (M_r_ = 548.05): C, 59.17; H, 4.41; N, 10.22%. Found: C, 59.29; H, 4.54; N, 10.21%. ESI + MS (MeOH, *m*/*z*): 661.04 (calc. 661.11) [Cu^I^_2_(L)_2_ + 3H]^+^, 485.10 (calc. 485.12) [Cu(bpy)(L)]^+^, 219.02 (calc. 219.00) [Cu^I^(bpy)]^+^. Λ_m_ (MeCN/MeNO_2_, 25 °C, S cm^2^ mol^−1^): 140/77 (electrolyte 1:1). IR peaks (ν_ATR_/cm^−1^): 3071 w, 3009 w, 2915 m, 2855 w, 2820 w, 1598 s, 1523 m, 1497 vs., 1465 m, 1437 m, 1369 s, 1308 s, 1177 s, 1146 s, 1026 s, 973 m, 857 w, 813 w, 756 m, 722 m, 651 m, 570 w, 5505 w. UV–Vis (MeCN/MeNO_2_/nujol): λ_max_ (nm): 276, 314, 444/440/282, 320, 426, 540. *µ*_eff_/*µ*_B_ (295 K): 1.86.

[Cu(dpa)(L)]NO_3_ (**6**). Yield: 64%. Anal. calcd. for C_27_H_25_N_5_O_5_Cu (M_r_ = 563.06): C, 57.59; H, 4.48; N, 12.44%. Found: C, 57.46; H, 4.73; N, 12.15%. ESI + MS (MeOH, *m*/*z*): 597.15 (calc. 597.18) [Cu^I^(L)_2_ + 2H]^+^, 500.18 (calc. 500.13) [Cu(dpa)(L)]^+^, 234.06 (calc. 234.01) [Cu^I^(dpa)]^+^. Λ_m_ (MeCN/MeNO_2_, 25 °C, S cm^2^ mol^−1^): 123/77 (electrolyte 1:1). IR peaks (ν_ATR_/cm^−1^): 3407 w, 3304 w, 3250 w, 3203 w 3141 w, 3040 w, 2909 w, 1642 m, 1590 s, 1504 s, 1480 s, 1429 m, 1400 m, 1359 m, 1301 s, 1178 m, 1149 s, 1022 m, 964 m, 807 w, 758 s, 715 w, 648 w, 571 w, 426 w. UV–Vis (MeCN/MeNO_2_/nujol): λ_max_ (nm): 254, 314, 440/440/430. *µ*_eff_/*µ*_B_ (295 K): 1.84.

### 2.2. Geometry Optimizations of Complex ***2*** Using DFT Calculations

The geometries of one of the most biologically relevant complex (i.e., complex (**2**)), both in the form involving a monodentate coordinated nitrato ligand (as expected in the solid state) [Cu(bphen)(L)(NO_3_)] and in the form of the cationic [Cu(bphen)(L)]^+^ complex containing ionic NO_3_ (as expected in solutions), were optimized using density functional theory (DFT) calculations at the ωB97X-D/def2-tzvp level of theory with the aim to not only visualize the most probable geometries of the compounds, but also to find the structural and geometrical differences between them. The calculations were performed using Spartan’20 [9] in vacuum and in the water solvation model. The figures were drawn, and additional structural calculations were performed using Mercury (ver. 3.9) software [10]. The selected geometrical parameters of both species are given in Table 1.

### 2.3. Single Crystal X-ray Analysis of [Cu(phen)(L)(NO_3_)]

The complex [Cu(phen)(L)(NO_3_)] was prepared and its composition was characterized as described elsewhere in the literature [8]. Crystals suitable for single crystal X-ray analysis were obtained by recrystallisation of the sample from DMF. The single crystal X-ray diffraction data of [Cu(phen)(L)(NO_3_)] were collected on a Bruker D8 Quest diffractometer equipped with a Photon 100 CMOS detector using Mo-Kα radiation at 293(2) K. Data collection, data reduction, and cell parameter refinements were performed using the Bruker Apex III software package [11]. The structure was solved by direct methods (SHELXS) and all non-hydrogen atoms were refined anisotropically on *F*^2^ using the full-matrix least-squares procedure in SHELXL-2014 [12]. Hydrogen atoms were found in differential Fourier maps and their parameters were refined using a riding model. Molecular graphics were prepared by Diamond [13] and some structural features were evaluated and interpreted using Mercury, ver. 3.9 [10]. The crystal data and structure refinements are given in Table 1.

### 2.4. Biological Experiments: Cell-Based Studies

#### 2.4.1. In Vitro Cytotoxicity

The standard MTT cell viability assay was used for the determination of the in vitro cytotoxicity. Three independent experiments using the cells within the 6th–15th passage, were carried out. The cells (ca. 25 000 cells per mL) were seeded in 96-well plastic microtitration plates and further pre-incubated at 37 °C in a CO_2_ atmosphere for 24 h. The complexes were dissolved in DMF (final concentration in medium 0.1% *v*/*v*) and applied to the cancer cells for 24 h. The MTT analysis was performed spectrophotometrically (TECAN, Männedorf, Switzerland) at 540 nm. The half-maximal inhibition concentrations IC_50_ were calculated where appropriate (see Supplementary Materials Figure S1). 

The following human cancer cell lines were used for cytotoxicity testing: ovarian carcinoma (A2780, Sigma (St. Louis, MO, USA), 93112519 and cisplatin resistant line A2780R, Sigma (St. Louis, MO, USA), 93112517), cervix epithelioid carcinoma (HeLa, ATCC CRM-CCL-2), prostate carcinoma (22Rv1, ATCC CRL-2505, and PC-3, ATCC CRL-1435), breast adenocarcinoma (MCF-7, ATCC HTB-22), human liver (HepG2, ATCC HB-8065), lung carcinoma (A549, ATCC CRM-CCL-185), human osteosarcoma (HOS, ATCC CRL-1543), and malignant melanoma (G361, ATCC CRL-1424). To assess the selectivity of the complexes, the normal human fetal lung fibroblasts (MRC-5, ATCC CCL-171) were used as a reference. The indicated cell lines were obtained from the American Type Culture Collection (ATCC, Manassas, VI, USA), and European Collection of Authorized Cell Cultures (ECACC, Salisbury, UK, sold by Sigma, St. Louis, MO, USA)), respectively, and were cultivated in Dulbecco’s modified Eagle medium (DMEM) containing 5 g/L glucose, 2 mM l-glutamine, 100 U/mL penicillin, 100 µg/mL streptomycin, 10% fetal calf serum, and sodium bicarbonate. Fetal lung fibroblast (MRC-5) cells were used as healthy cells in the case of complex **2**, showing the overall lowest IC_50_ values.

#### 2.4.2. Cell Culture for Molecular Biology Assays

For the study of the mechanism of the in vitro cytotoxicity analysis, the human ovarian cancer cell line A2780 (Sigma (St. Louis, MO, USA), 93112519) was used. When performing the experiments, the cells were kept at 37 °C with a 5% CO_2_ atmosphere in an incubator and cultured in complete RPMI-1640 medium (Sigma Aldrich, St. Louis, MO, USA); the final concentrations of supplements were as follows: L-glutamine (2 mM), fetal bovine serum (FBS, 10%) and PenStrep (5 U penicillin, 50 µg streptomycin/mL). Complex **2**, as the most promising candidate as for the cytotoxicity experiments, was chosen for deeper biological studies.

#### 2.4.3. Cell Cycle Analysis

To study the potential changes in the cell cycle of A2780 cells after treatment with complex **2**, we seeded 10^4^ cells/well in a 96-well plate. The next day, the cells were treated with 3 µM of complex **2** and incubated for 24 h. As a reference drug, the platinum-based metallotherapeutic cisplatin was used in a 19 µM concentration, corresponding to the IC_50_ value from the cytotoxicity testing after 24 h of incubation. Then, the supernatant was discarded and after a washing step with PBS (0.1 M, pH 7.4), cell cycle analysis using the BD Cycletest^TM^ Plus DNA Kit (Becton Dickinson, Franklin Lakes, NJ, USA) was performed according to the manufacturer’s protocol. Every experiment was conducted in duplicate on a BD FACSVerse flow cytometer (Becton Dickinson, Franklin Lakes, NJ, USA), where at least 5 × 10^3^ events were acquired for each sample.

#### 2.4.4. Apoptosis, Autophagy, and Mitochondrial Membrane Potential (ΔΨ m) Analyses

To determine the effect of complex **2** on the apoptosis, autophagy, and ΔΨm of A2780 cells, flow cytometry experiments were realized. Briefly, we seeded 5 × 10^4^ cells/well in 24-well plates and left to let the cells adhere. The next day, we treated cells with 2 µM of complex **2** and incubated it for 24 h. Then, we collected the supernatant, washed cells once with PBS (0.1 M, pH 7.4), used trypsin (0.25% sterile water solution, containing 0.2 g/L of ethylenediaminetetraacetic acid (EDTA), Sigma-Aldrich, St. Louis, MO, USA) to detach them, and made a 500 µL suspension in culture media. Finally, we stained the samples with appropriate dyes for the specific assay and analyzed them using a BD FACSVerse flow cytometer (Becton Dickinson, Franklin Lakes, NJ, USA). Each experiment was performed in duplicate and at least 5 × 10^3^ events were recorded for each sample.

For the apoptosis analysis, two different assays were selected. This is why the sample for this experiment was divided into two separate tubes for either apoptosis or caspase activity determination. The determination of caspases 3/7 induction was conducted by the CellEvent^TM^ Caspase-3/7 Green Flow Cytometry Assay Kit (Thermo Fisher Scientific, Waltham, MA, USA), while for apoptosis, propidium iodide (PI) and Annexin V-FITC apoptosis detection kits were selected (Enzo Life Sciences, Farmingdale, NY, USA). The apoptosis assay was conducted according to the manufacturer’s protocol, while the caspases 3/7 experiment was modified, as only the CellEvent^TM^ Caspase-3/7 Green Detection Reagent for the detection of caspase-3/7 activation was used. The MITO-ID^®^ Membrane Potential Detection Kit (Enzo Life Sciences, Farmingdale, NY, USA) and CYTO-ID^®^ Autophagy Detection Kit 2.0 (Enzo Life Sciences, Farmingdale, NY, USA) were chosen for the ΔΨm and autophagy analysis, respectively. Both types of experiments were performed precisely according to the manufacturers’ protocols.

#### 2.4.5. Reactive Oxygen Species (ROS) Production Determination

For the ROS production analysis, the modified version of the ROS-ID^®^ Total ROS/Superoxide Detection Kit (Enzo Life Sciences, Farmingdale, NY, USA), where only the total ROS production measurement according to the manufacturer’s protocol was performed. The A2780 cells were seeded at the concentration of 10^4^ cells/well to a 96-well plate and consecutively treated either with 3 µM of complex **2**, or 19 µM of cisplatin. The samples were prepared with or without the addition of 100 µM of pyocyanin (a known pro-oxidative agent used as positive reference standard), incubated for 24 h, and then washed with PBS (0.1 M, pH 7.4). The fluorescence of samples was measured in triplicate using a multimode microplate reader Infinite PRO M200 (TECAN, Männedorf, Switzerland).

### 2.5. Cellular Uptake of Copper in A2780 and 22Rv1 Cancer Cells

A number of 10^6^ A2780 and 22Rv1 cells were treated with complex **2** at a 3 µM concentration for 2, 6, 12, 24, 48, and 72 h. After treatment, the cells were detached by trypsinization, washed with PBS (0.1 M, pH 7.4), and collected by centrifugation. The cell pellets were treated with 500 µL of concentrated nitric acid for ICP-MS (TraceCERT^®^, Supelco, Wajtham, MA, USA) and heated to 70 °C for 24 h. The intracellular concentration of copper in the cancer cells was determined by means of ICP-MS (Agilent 7700x, Agilent Technologies, Santa Clara, CA, USA) using the external calibration (Multielement standard solution 1 for ICP, Supelco, Waltham, MA, USA).

### 2.6. Interactions with Model Proteins

Two model proteins, bovine serum albumin (BSA) and horseradish peroxidase, were each (100 nmol) reduced in 50 mM dithiothreitol (DTT) in 20 potassium phosphate, pH 7.0 (working buffer), at 37 °C overnight. The reagent was removed by chromatography on Sephadex G-25. Aliquots of the reduced and chromatographed proteins (2 nmol) were mixed with 1 mL of 100 µM solution of complex **2** in the working buffer and incubated at 37 °C overnight. The mixture was then separated on the Sephadex G-25 column in 50 NH_4_HCO_3_ adjusted to pH 7.0. The collected protein material was recovered by evaporation in a vacuum centrifuge and reconstituted in 15 µL of 0.1% trifluoroacetic acid for subsequent purification using ZipTip-C4 pipette tips (Merck-Millipore, Tullagreen, Ireland) according to the manufacturer’s instructions. Matrix-assisted laser desorption/ionization time-of-flight mass spectrometry (MALDI-TOF MS) was then applied to determine the intact protein molecular mass values as previously described [14]. In an additional experiment, discontinuous native electrophoresis [15] of BSA and rabbit glycogen phosphorylase B proteins was run at 120 V using a 12% separating polyacrylamide gel containing 100 µmol/L complex **2** and their relative mobility compared with that in a standard gel. The separated proteins were then subjected to an in-gel digestion procedure followed by MALDI-TOF peptide mass fingerprinting [16].

### 2.7. Statistical Evaluation

For the flow cytometry experiments and total ROS determination, three independent experiments were conducted and the mean ± standard deviations (SD) were calculated. One-way ANOVA with the Tukey post-hoc test was used in Statistica software, ver. 13 [17] and a significant difference compared to the negative control was highlighted. Differences were considered significant at *p* ≤ 0.05 (*), *p* ≤ 0.01 (**), and *p* ≤ 0.001 (***).

## 3. Results and Discussion

### 3.1. Synthesis and Characterization of the Complexes

Complexes **1**–**6** were prepared by a slightly modified procedure as described in the literature [8] as follows: complexes **1**–**6** were prepared by the reaction with the corresponding aromatic diimine ligand (0.5 mmol) and HL ligand (0.5 mmol) in MeOH:CH_2_Cl_2_ (1:1, *v*/*v*). Then, the equimolar amount of triethylamine was added during stirring. After, a solution of Cu(NO_3_)_2_·3H_2_O (0.5 mmol) in 5 mL of MeOH was added. The reaction mixture was stirred under reflux for 30 min, subsequently filtered and left to stand to evaporate at laboratory temperature. The solid phase, which formed after a few days, was filtered off, washed by a cold MeOH (2 × 5 mL), and dried in a vacuum desiccator over solid NaOH. The composition of the complexes was determined by various techniques including elemental analysis, infrared and electronic spectroscopies, mass spectrometry, magnetic susceptibility, and conductivity measurements. Single-crystal X-ray analysis of [Cu(phen)(L)(NO_3_)] revealed a structure of the structurally similar complex [Cu(bphen)(L)(NO_3_)] (**2**). The IR spectra of complexes **1**–**6** showed typical peaks [18] belonging to the corresponding bidentate *N*-donor aromatic diimine and L ligands (in cm^−1^): 3000–3100 ν(C–H)_arom._, 2800–3000 ν(C–H)_aliph_, 1590–1598 ν(C–N)_arom,_, *ca.* 1520, and 1485 ν(C–C)_arom_., *ca.* 1430 ν_5_(NO), *ca.* 1310 ν_1_(NO), and *ca.* 1026 ν_2_(NO) in an unidentate coordination of NO_3_ ligand, and 675–900 δ(C–H) cm^−1^. The IR spectra of complexes **1–6** are shown in the Supplementary Materials as Figures S2–S7. The electronic spectra of complexes **1**–**6** were measured in both the solid state (Nujol mulls) and in solution (MeCN and MeNO_2_). They showed a maxima of charge transfer transitions in the region of 420–450 nm in solution and solid-state, and *d*–*d* transitions at ca. 518–557 nm in the solid-state spectra (see Figures S8–S13 in Supplementary Materials). The spectra support the structural similarity of the complexes in the solid state and in the solvents used (MeCN and MeNO_2_). The peaks corresponding to the [Cu(L)(N–N)]^+^ complex species were identified in the mass spectra of the complexes. The mass spectra of complexes **1–6** are shown in Figures S14–S19 in the Supplementary Materials. The molar conductivity data showed that the complexes behaved as 1:1 electrolytes in MeCN and MeNO_2_ [19] (see Table S1 in Supplementary Materials). The values of the effective magnetic moments (*µ*_eff_/*µ*_B_ = 1.74–1.86) of complexes **1**–**6** lie within the typical interval for copper(II) ions in a d^9^ configuration with one unpaired electron [20].

### 3.2. Geometry Optimization Using DFT Calculations

**Figure 1:**
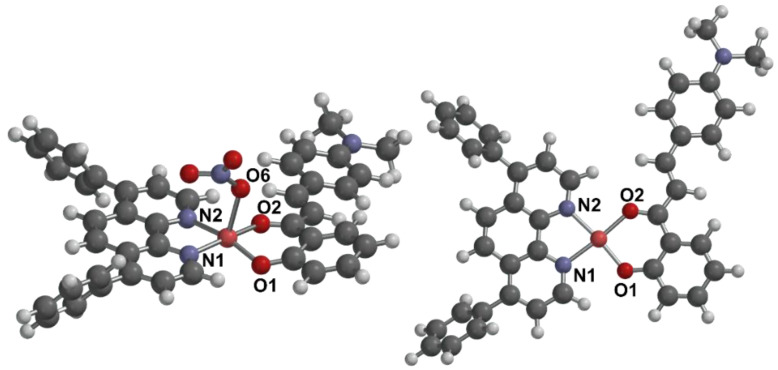
The geometries of the [Cu(bphen)(L)(NO_3_)] (**2**) (**left**) and [Cu(bphen)(L)]^+^ (**right**) species optimized in a water solvation model at the ϖB97X-D/def2-tzvp level of theory. The color coding of the atoms is as follows: hydrogen atom−light gray, carbon atom−dark gray, nitrogen atom−blue, oxygen atom−red, copper atom−orange.

Based on the fact that we were not able to prepare suitable single crystals for any of the complexes (**1**–**6**) for detailed structural characterization, although we performed many attempts and made every effort to achieve that, we decided to optimize a geometry of one of the most biologically relevant complexes (i.e., complex (**2**)), both in the form of a monodentate coordinated nitrato ligand (as expected in the solid state), [Cu(bphen)(L)(NO_3_)] and in the form of the cationic [Cu(bphen)(L)]^+^ complex (as expected in solutions). Surprisingly, we were successful in the preparation of single-crystals of the formerly published Cu–chalcone complex [8] with the composition of [Cu(phen)(L)(NO_3_)] involving 1,10-phenanthroline (phen) instead of 4,7-diphenyl-1,10-phenanthroline (bphen). Its detailed structural description is given in Section 3.3 in this paper. The two possible geometries of complex (**2**) were optimized using density functional theory (DFT) calculations at the ωB97X-D/def2-tzvp level of theory with the aim to visualize not only the most probably geometries of the compounds, but also in order to find the structural and geometrical differences between them (see Figure 1). The calculations were performed using Spartan’20 in the vacuum and in water solvation models [9] The figures were drawn using Mercury [10] software. Selected interatomic parameters of both the optimized molecular geometries are given in Table 2. As can be seen from the table, the selected interatomic parameters between both the DFT calculated geometries differed significantly in the vicinity of the copper(II) atoms, which can be associated dominantly with different polyhedra around the central metal atoms (a distorted square-pyramidal versus a distorted square-planar geometry).

**Figure 2:**
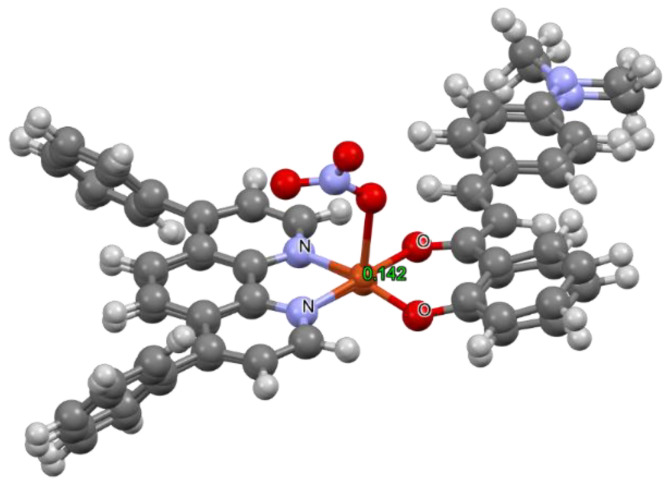
An overlay of the DFT optimized geometries of the [Cu(bphen)(L)(NO_3_)] (**2**) and [Cu(bphen)(L)]^+^ species in the water solvation model through the N_2_O_2_ donor atoms, showing the separation of the Cu(II) atoms of 0.142 Å. The color coding of the atoms is as follows: hydrogen atom−light gray, carbon atom−dark gray, nitrogen atom−blue, oxygen atom−red, copper atom−orange.

**Figure 3:**
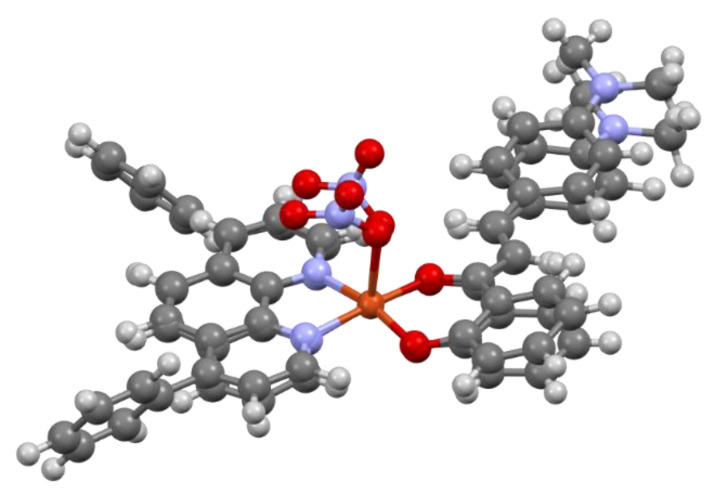
An overlay of the DFT optimized geometries of [Cu(bphen)(L)(NO_3_)] (**2**) (in the water solvation model) with that determined by means of X-ray analysis [Cu(phen)(L)(NO_3_)] through the N_2_O_2_ donor set. The color coding of the atoms is as follows: hydrogen atom−light gray, carbon atom−dark gray, nitrogen atom−blue, oxygen atom−red, copper atom−orange.

The similarities on one side and differences on the other side of both structures calculated using the water solvation model can be seen in Figure 2, which depicts an overlay of both molecules over the N_2_O_2_ donor atoms. As expected, the copper(II) atom was situated in the square-pyramidal geometry of [Cu(bphen)(L)(NO_3_)] (**2**) 0.142 Å above that in the square-planar geometry of [Cu(bphen)(L)]^+^. However, some of the interatomic parameters within the square-pyramidal polyhedra between the DFT calculated structure of [Cu(bphen)(L)(NO_3_)] and the X-ray determined structures of [Cu(phen)(L)(NO_3_)] [this work] and [Cu(phen)(chal1)(NO_3_)] [8] also differed significantly. Thus, we performed additional calculations using the water solvation model with the aim to create more realistic conditions influencing the final coordination geometry of the studied compounds. The results revealed that the selected interatomic parameters around the copper(II) atom between the DFT calculated geometry and those determined experimentally were very close to each other, and thus the calculations at the ϖB97X-D/def2-tzvp level of theory using the water solvation model provide meaningful results for the molecular geometry of [Cu(bphen)(L)(NO_3_)] (**2**). Moreover, the geometries of the optimized molecule [Cu(bphen)(L)(NO_3_)] (**2**) (in water solvation model) and that of the structurally characterized [Cu(phen)(L)(NO_3_)] were overlaid through the N_2_O_2_ donor set, as depicted in Figure 3, showing significant structural similarities of both molecules. The coordinates for the optimized geometries of the complex species are given in the Supplementary Materials (see Table S2) in the XYZ format.

### 3.3. Single-Crystal X-ray Analysis of [Cu(phen)(L)(NO_3_)]

**Figure 4:**
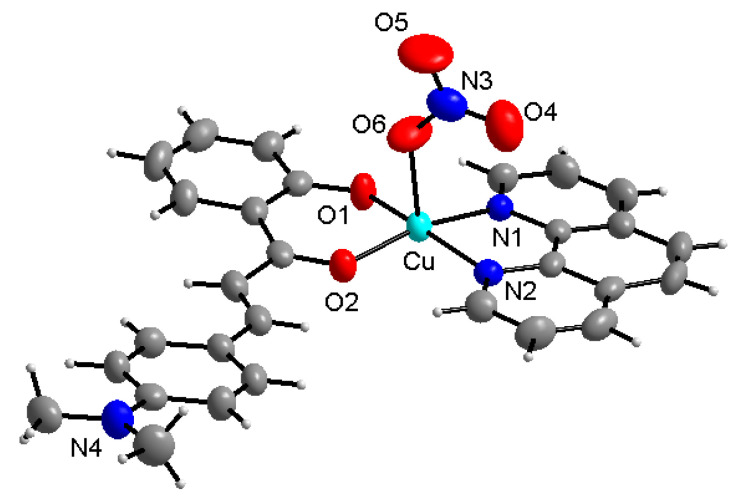
Molecular structure of [Cu(phen)(L)(NO_3_)] as determined by a single crystal X-ray analysis. The carbon atoms are depicted using a dark-grey color, while the H-atoms are depicted in a light-grey color. The color coding of the atoms is as follows: hydrogen atom−light gray, carbon atom−dark gray, nitrogen atom−blue, oxygen atom−red, copper atom−turquoise.

Our efforts to prepare single crystals of complexes **1**–**6**, which would be suitable for X-ray structural characterization, failed. However, we were successful in the preparation of single crystals of a good quality for the formerly published complex [Cu(phen)(L)(NO_3_)] [8], which contains 2′-hydroxy-4-(dimethylamino)chalcone (HL) and 1,10-phenanthroline (phen) as ligands in the coordination sphere of the copper(II) atom, and thus this complex may serve as a good candidate for the description of structural features of the herein presented complexes **1**–**6**. The molecular structure of the complex is depicted in Figure 4, while the selected interatomic parameters are given in Table 3. The geometry in the vicinity of the central Cu(II) atom can be ascribed as a spherical square pyramid, as calculated using the SHAPE program [21]. The results are shown in the Supplementary Materials in Table S3.

The crystal structure of [Cu(phen)(L)(NO_3_)] is stabilized by non-covalent contacts of the C–H···O, C–H···C and C···O type. Crystal packing, together with the selected non-covalent contacts, is depicted in the Supplementary Materials in Figure S20 and Table S4.

### 3.4. In Vitro Cytotoxicity of Complexes ***1**–**6***

The effectiveness of complexes **1**–**6** to influence the viability of cancer cells was studied via in vitro screening using the MTT assay on eight human cancer cell lines (HeLa, 22Rv1, MCF7, PC3, HepG2, HOS, A549, and G361). The results (Table 4) indicate that the highest effects on the metabolic activity of the cancer cells showed that complexes **1**–**3**, involved bulky disubstituted diimine ligands (5,5′-dimethyl-2,2′-bipyridine (**1**), bphen = 4,7-diphenyl-1,10-phenanthroline (**2**), and dbbpy = 4,4′-di-*tert*-butyl-2,2′-bipyridine (**3**)) in the structure of their complex cations. These findings correlate with the conclusions of several published works regarding the higher affinity of copper(II) complexes involving bulky substituted diimine ligands to DNA binding and cytotoxicity [22] and increased nuclease activity [23]. This hypothesis is also supported by our previous studies involving the mixed-ligand copper(II) complexes involving the natural or nature-inspired ligands in combination with derivatives of 1,10-phenanthroline or 2,2′-bipyridine [24,25]. Complexes **1–3** showed a wide-spectrum effect on all cancer cell lines (except for complex **1** on PC3 cell line), exceeding the cytotoxicity of the reference drug cisplatin in all cases. The most potent complex **2** showed more than 10-times higher effectiveness compared to cisplatin in most of the cases and therefore was chosen for further biological studies. Complex **2**, as the compound with the overall lowest IC_50_ values against cancer cells, was chosen as the representative sample for determination of the in vitro toxicity on lung fibroblasts (MRC-5) as healthy cells to reveal the selectivity of the most cytotoxic complex. It was not possible to determine the toxicity within the concentration range of 0.1–50 μM (the highest concentration limit due to the limited solubility in the cultivation medium), and thus the inhibition concentration can be expressed as IC_50_ > 50 μM. This finding clearly showed a relative low toxicity of complex **2** to healthy cells compared to the cancer cells used, and thus the significant selectivity of the compounds.

### 3.5. Effect of Complex ***2*** on the Cell Cycle of A2780 Cells

**Figure 5:**
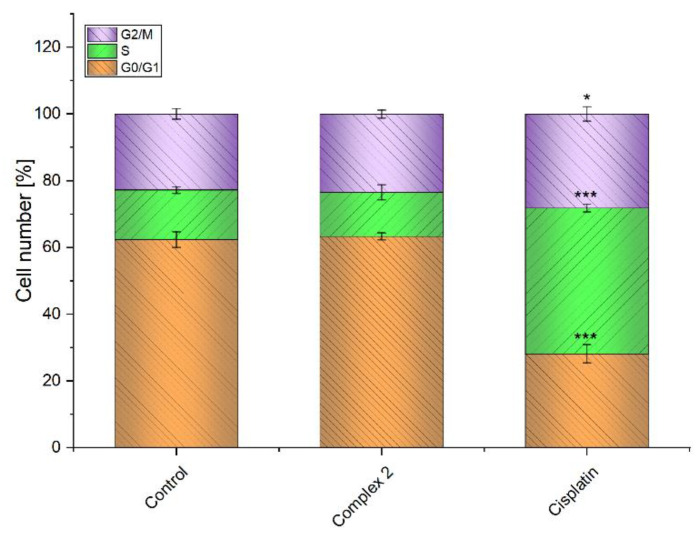
Cell cycle modifications of the A2780 cells induced by complex **2** (applied at 3 µM concentration) and cisplatin (applied at 19 µM concentration) after 24 h of incubation. The significant change in the number of cells in a specific phase of the cell cycle caused by the respective complex compared to the untreated control at different significance levels is represented by * *p* ≤ 0.05, and *** *p* ≤ 0.005.

The cellular effects of the overall best performing complex **2** were studied on the A2780 cell line at the concentration corresponding to the IC_50_ value obtained from the 24 h cytotoxicity test. In contrast to cisplatin (see Figure 5 and Figure S21 in the Supplementary Materials), used as the reference metal-based chemotherapeutic agent, which significantly increased the number of cells arrested in the S-phase of the cell cycle (43.6 ± 1.2%) and the G2/M cell-phase (28.2 ± 1.2% vs. 22.8 ± 1.5% in untreated cells), complex **2** caused no significant changes in the cell cycle of the A2780 cells compared to the untreated control (portion of cells in S-phase was 14.8 ± 1.0%, and 13.2 ± 2.2% in the case of the untreated control and complex **2** treated cells, respectively). These findings, together with the antiproliferative screening data, suggest that significant differences exist in the mechanisms of action between the studied copper(II) complex **2** and the platinum-based chemotherapeutic drug cisplatin.

### 3.6. Induction of Apoptosis and Associated Processes

**Figure 6:**
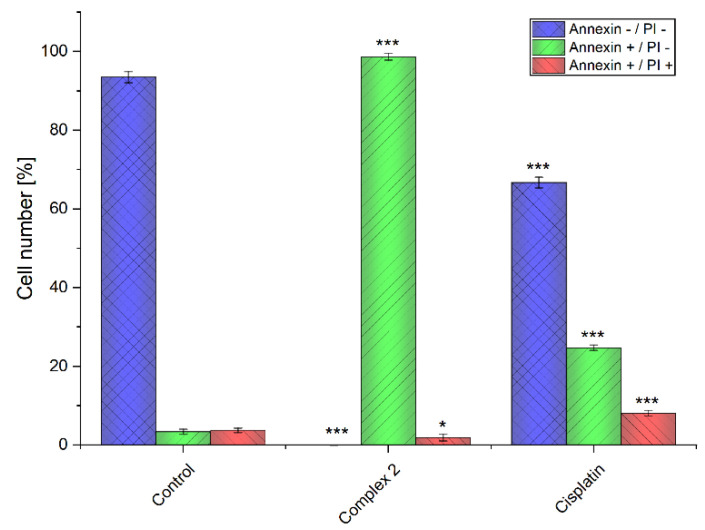
The effects of complex **2** (3 μM) and cisplatin (19 μM) on cell death induction in the A2780 cells after 24 h of incubation. The significant change in the number of cells in different stages of cell life/death caused by the respective complex compared to the untreated control is symbolized by * *p* ≤ 0.05, and *** *p* ≤ 0.005 level.

The effects of the most cytotoxic complex **2** and the reference drug cisplatin on the induction of cell death were studied by means of flow cytometry in the A2780 cells after 24 h of incubation with the tested compounds (see Figure 6 and Figure S22 in the Supplementary Materials). The effects of both the tested compounds differed significantly and while cisplatin induced cell death in about 33% of the cells (while 8% of cells reached the late-stage apoptosis), complex **2** caused the transition from normal cellular metabolism to the state with Annexin V positive staining of the cellular membranes, a condition considered specific for early stages of apoptosis in ca. 99% of A2780 cells.

**Figure 7:**
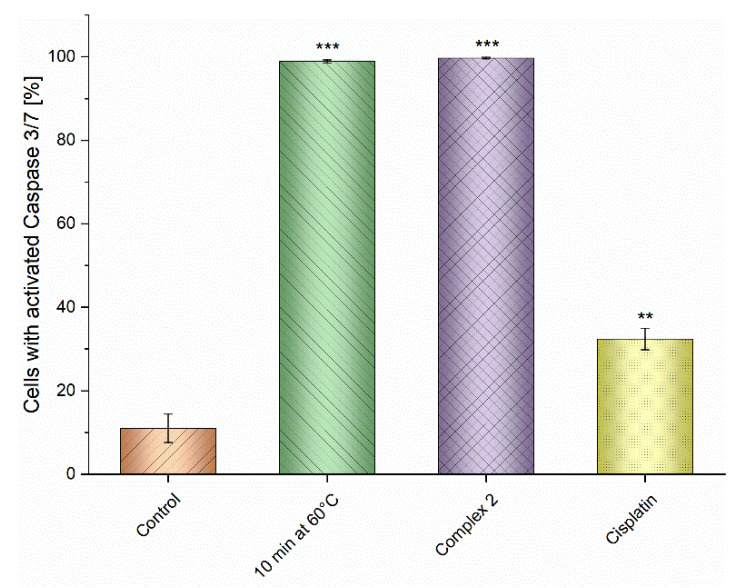
Activation of caspases 3/7 by complex **2** (applied at 3 µM) and cisplatin applied to the A2780 cells at a 19 µM concentration after 24 h of incubation. The significant change in the number of cells with activated caspases 3/7 compared to the untreated control is symbolized by ** at *p* ≤ 0.01 level, and *** at *p* ≤ 0.005 level.

The above-mentioned findings regarding the strong ability of complex **2** to promote the transition of A2780 cells into apoptosis were also confirmed by the determination of the activated executioner caspases 3/7 in the A2780 cells (see Figure 7 and Figure S23 in the Supplementary Materials). Complex **2** induced the activation of caspases 3/7 in practically all of the cells.

### 3.7. Mitochondrial Membrane Potential Disruption

**Figure 8:**
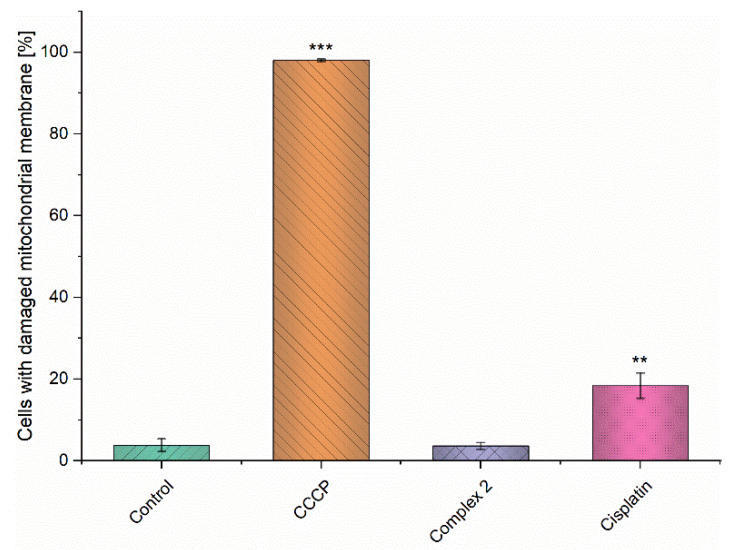
Effect of complex **2** (at 3 µM), reference drug cisplatin (at 19 µM), and the well-known inhibitor of mitochondrial oxidative phosphorylation carbonyl cyanide *m*-chlorophenyl hydrazone CCCP (100 µM) on the mitochondrial membrane potential disruption in A2780 cells after 24 h of incubation. *** indicates statistical significance at the *p* < 0.005 level and ** *p* < 0.01 levels compared to the control.

In order to better understand the mechanisms leading to apoptosis in the A2780 cells caused by complex **2**, we performed the determination of mitochondrial membrane potential disruption by means of flow cytometry in the A2780 cells after 24 h of incubation (see Figure 8 and Figure S24 in the Supplementary Materials). Interestingly, in contrast to cisplatin, the complex **2** treatment had no effect on mitochondrial metabolism and mitochondrial membrane integrity in the A2780 cells, which might indicate that complex **2** acts as an inductor of the extrinsic apoptosis pathway, mediated by the activation of cell membrane death receptors such as FasR, TRAIL, or TNF-α receptors [26].

### 3.8. Intracellular Production of Reactive Oxygen Species (ROS) and Induction of Autophagy

**Figure 9:**
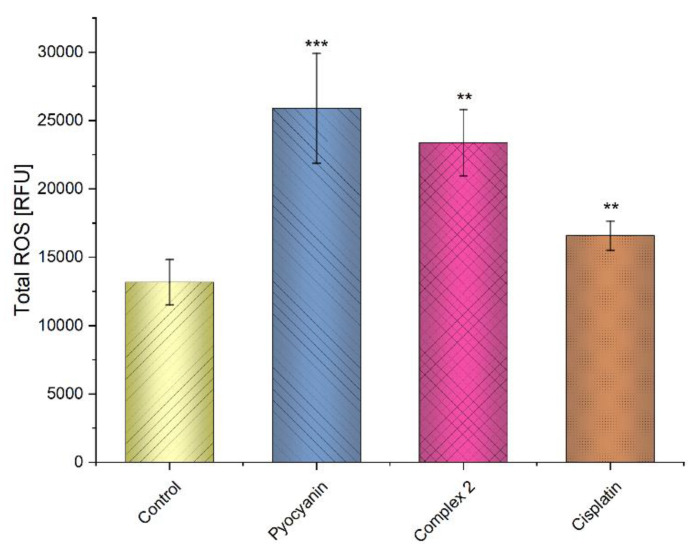
The effects of complex **2** (3 µM), reference drug cisplatin (19 µM), and well-known inductor of ROS production pyocyanin (100 µM) on the total ROS production in the A2780 cell line. Cells were treated by the tested compounds, or by the vehicle (0.1% DMF) in the control sample for 1 h. *** indicates statistical significance at the *p* < 0.005 level and ** *p* < 0.01 levels compared to the control.

Reactive oxygen species (ROS) play a crucial role in the cellular metabolism of the cells [27]. While moderate levels of ROS are considered as an environmental stressor, high levels of ROS are one of the preconditions of cell death [28]. Our results (see Figure 9) indicate that complex **2** is a strong elicitor of oxidative stress in A2780 cells. These findings indicate that this might be the main molecular mechanism leading to the cell death in cancer cells.

**Figure 10:**
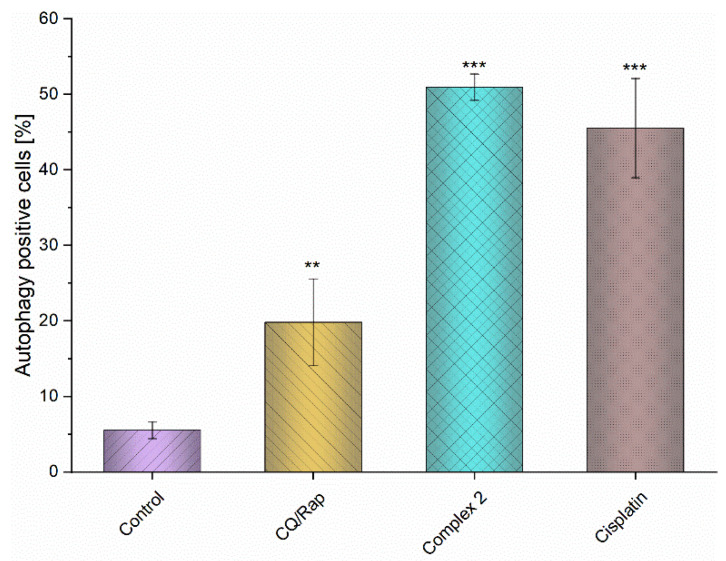
The induction of autophagy in the A2780 cells caused by complex **2** (3 µM), reference drug cisplatin (19 µM), and the mixture of chloroquine and rapamycin, a moderate inductor of autophagy after 24 h of incubation. The significant change in the number of cells showing the signs of autophagy caused by the corresponding compound compared to the untreated control is symbolized by ** at the *p* ≤ 0.01 level, and *** at the *p* ≤ 0.005 level.

Intracellular oxidative stress is a known cause of deleterious damage to vital molecules of life (such as nucleic acids, lipids, and proteins [29]) and as an inducer of both apoptosis and autophagy [30]. In this context, we strived to investigate the ability of complex **2** to induce the signs of autophagy in the A2780 cells. For this screening, we utilized the CYTO-ID^®^ Autophagy Detection Kit 2.0, which is able to identify the presence of autophagic vacuoles and monitor the autophagic flux in lysosomally inhibited live cells using a specific dye that selectively labels accumulated autophagic vacuoles (pre-autophagosomes, autophagosomes, and autolysosomes). Indeed, complex **2** proved to also be an efficient inductor of autophagy in the A2780 cells (see Figure 10 and Figure S25 in the Supplementary Materials). These findings indicate that the cellular effects of complex **2** are multimodal, targeting various cellular processes in the A2780 cells. A similar biological profile was described in the copper(II) complex involving the di-2-pyridyl ketone 4-allyl-3-selenosemicarbazide ligand (designated as HYF127c/Cu), which proved to be an efficient inductor of oxidative stress, and consequently, both apoptosis and autophagy in HeLa cells by activating the MAPK11/12/13/14 (p38 MAPK) pathway [31].

### 3.9. Accumulation of Copper in A2780 and 22Rv1 Cancer Cells

The intracellular transport of complex **2** to A2780 cells was studied by the ICP-MS method in the most susceptible A2780 cells and 22Rv1 cells in six time periods from the moment of the addition of complex **2** into the cultivation media, namely, after 2, 6, 12, 24, 48, and 72 h (see Figures S26 and S27 in the Supplementary Materials). In both cell lines, the levels of intracellular copper did not differ significantly from the untreated control cells at all times used. This is not a surprising finding as the studied copper(II) complexes **1**–**6** behave as ionic complexes containing the bulky complex cations in solutions. These results also support the finding that the cytotoxicity against cancer cells is mediated via the extrinsic mechanism of apoptosis induction by the interaction of complex **2** with the key elements of cellular membranes.

### 3.10. Interactions with Model Proteins

MALDI-TOF MS was applied to find out whether complex **2** could modify proteins. BSA and horseradish peroxidase in their reduced forms (a maximum of free thiol groups was thus available) were chosen as model soluble proteins. Their aliquots were incubated with the compound, chromatographed, and then subjected to MALDI-TOF MS analyses, showing no significant difference in the molecular mass toward the controls (66.5, and 43.2 kDa, respectively). BSA was additionally separated by native polyacrylamide gel electrophoresis in the presence of 100 µM complex inside the separating gel. No mobility shift of the protein band of BSA was registered after Coomassie-based visualization when compared with the control gel without complex **2**. The same result was obtained for rabbit glycogen phosphorylase B (GPB). The protein material separated by electrophoresis was subjected to an in-gel digestion procedure and the generated tryptic peptides were analyzed by MALDI-TOF MS. The peptide spectra of the control and sample separated were found to be practically identical with regard to the representation of peaks assigned to the amino acid sequence. All of these results are given in the Supplementary Materials in Figure S28. Such observations seem to exclude any possible covalent binding of the complex compound **2** to proteins, and indicate rather that noncovalent interactions may occur.

## 4. Conclusions

A series of six heteroleptic copper(II) complexes, containing a combination of nature inspired 2′-hydroxy-4-(dimethylamino)chalcone (HL) ligand and bidentate aromatic *N,N*-donor imine ligand (N-N), of the general composition [Cu(N-N)(L)]NO_3_ (**1**–**6**), was prepared. The complexes were thoroughly characterized using different techniques to clearly support their composition and purity. The MTT method-based screening of in vitro cytotoxicity was performed against eight human cancer cell lines (HeLa, 22Rv1, MCF-7, PC3, HepG2, A549, HOS, A549, and G361), which revealed that complexes **1**, **2**, and **3** involving bulky *N–N* ligands act as effective antiproliferative agents, with the best performing complex **2**, containing the bathophenanthroline (bphen) ligand, showing IC_50_ values in the range of 1.0–2.3 μM. The best performing complex **2** also revealed good selectivity toward the cancer cells as its toxicity on healthy fetal lung fibroblasts MRC-5 was low, with an IC_50_ higher than 50 μM. The chemical interactions of complex **2** with the selected model proteins were studied by means of MALDI-TOF and chromatographic methods, showing its limited ability to bind covalently to the protein backbone. Following the cytotoxicity screening, the biological effects of complex **2** on the cellular metabolism of A2780 cancer cells (cell cycle, cell death induction, caspases 3/7 activation, mitochondrial membrane permeation, ROS generation, and autophagy induction) were performed. These cell-based assays revealed the strong pro-apoptotic effects of complex **2**, which was able to effectively activate the executioner caspases 3/7, ROS overproduction, and autophagy in the A2780 cells, while not impeding the cell cycle and mitochondrial membrane functions. The cellular uptake of complex **2** in the A2780 and 22Rv1 cells studied by the ICP-MS method revealed no significant transport of the cationic complexes into the cancer cells, supporting the hypothesis that the in vitro anticancer effects of complex **2** are based on concerted extrinsic activation of apoptosis and autophagy induction.

## Figures and Tables

**Table 1:** Crystal data and structure refinement for [Cu(phen)(L)(NO_3_)].

Molecular formula	C_29_ H_24_ Cu N_4_ O_5_
Formula weight	572.06
Temperature	295(2) K
Wavelength	0.71073 Å
Crystal system	Monoclinic
Space group	*P*2**_1_**/n
Unit cell dimensions	a = 10.454(4) Å, α= 90°
	b = 16.197(5) Å, β= 96.95(2)°
	c = 14.748(5) Å, γ = 90°
Volume	2478.8(15) Å^3^
*Z*	4
Density (calculated)	1.533 g/cm^3^
Absorption coefficient	0.931 mm^−1^
*F*(000)	1180
Crystal size	0.200 × 0.180 × 0.160 mm^3^
*θ* range for data collection	2.26 to 24.82°
Index ranges	−12 ≤ *h* ≤ 12, −19 ≤ *k* ≤ 19, −17 ≤ *l* ≤ 17
Reflections collected	23,346
Independent reflections	4255 [*R*(int) = 0.1006]
Completeness to *θ* = 24.821°	99.30%
Absorption correction	Multi-Scan method (SADABS)
Refinement method	Full-matrix least-squares on *F*^2^
Data/restraints/parameters	4255/0/352
Goodness-of-fit on *F*^2^	1.051
Final *R* indices [I > 2σ(I)]	*R*1 = 0.0449, w*R*2 = 0.0821
*R* indices (all data)	*R*1 = 0.0869, w*R*2 = 0.0957
Largest diff. peak and hole	0.312 and −0.308 e. Å^−3^

**Table 2:** Selected interatomic parameters (in Å and °) in the vicinity of the copper(II) atom for both forms of complex (**2**) as determined using DFT calculations at the ωB97X-D/def2-tzvp level of theory in the vacuum and water solvation models, and their comparison with the X-ray structurally determined values for [Cu(phen)(L)(NO_3_)] and [Cu(phen)(chal1)(NO_3_)], where Hchal1 = 2′-hydroxychalcone {(E)-1-(2′-hydroxyphenyl)-3-phenylprop-2-en-1-one}.

Selected Interatomic Parameters	In Vacuum	In Water	In Vacuum	In Water	X-ray ^#^	X-ray *
	[Cu(bphen)(L)(NO_3_)] ϖB97X-D/def2-tzvp		[Cu(bphen)(L)]^+^ ϖB97X-D/def2-tzvp		[Cu(phen)(L)(NO_3_)]	[Cu(phen)(chal1)(NO_3_)]
Cu–O1	1.906	1.903	1.857	1.881	1.872 (2)	1.8839 (18)
Cu–O2	1.959	1.949	1.907	1.926	1.924 (2)	1.9260 (18)
Cu–O6	2.159	2.358	-	-	2.341 (3)	2.429 (2)
Cu–N1	2.035	2.018	2.016	2.014	2.004 (3)	2.001 (2)
Cu–N2	2.050	2.025	2.025	2.015	2.004 (3)	1.995 (2)
N1–Cu–O2	164.14	171.82	174.25	174.76	166.40 (11)	173.51 (8)
N2–Cu–O1	162.44	167.51	172.26	173.10	168.54 (11)	168.68 (9)
N1–Cu–O6	103.28	96.87	-	-	105.85 (12)	95.29 (8)
O1–Cu–O6	102.02	93.33	-	-	96.49 (11)	91.61 (8)

^#^ Data published in this work, see Section 3.3. * Data adopted from ref. [8].

**Table 3:** Selected bond lengths [Å] and angles [°] for [Cu(phen)(L)(NO_3_)].

Bond Length [Å]		Angle [°]	
Cu(1)-O(1)	1.872 (2)	O(1)-Cu(1)-O(2)	91.69 (10)
Cu(1)-O(2)	1.924 (2)	O(1)-Cu(1)-N(2)	168.54 (11)
Cu(1)-N(2)	2.004 (3)	O(2)-Cu(1)-N(2)	94.67 (11)
Cu(1)-N(1)	2.004 (3)	O(1)-Cu(1)-N(1)	89.51 (11)
Cu(1)-O(6)	2.341 (3)	O(2)-Cu(1)-N(1)	166.40 (11)
N(3)-O(5)	1.223 (4)	N(2)-Cu(1)-N(1)	82.09 (11)
N(3)-O(4)	1.230 (4)	O(1)-Cu(1)-O(6)	96.49 (11)
N(3)-O(6)	1.239 (4)	O(2)-Cu(1)-O(6)	87.48 (11)
		N(2)-Cu(1)-O(6)	93.30 (11)
		N(1)-Cu(1)-O(6)	105.85 (12)

**Table 4:** The in vitro cytotoxicity of complexes **1–6** and the reference drug cisplatin after 24 h of incubation. The data are expressed as IC_50_ ± SD (μM) calculated from the dose–response curves obtained from at least three independent determinations.

Compound	Human Cancer Cell Lines							
	HeLa	22Rv1	MCF7	PC3	HepG2	HOS	A549	G361
**1**	3.3 ± 0.2	7.9 ± 0.6	13.7 ± 1.8	>50	16.5 ± 0.3	5.2 ± 0.8	14.9 ± 2.6	6.9 ± 0.5
**2**	2.0 ± 0.1	1.0 ± 0.1	1.6 ± 0.1	2.2 ± 0.2	2.3 ± 0.5	2.1 ± 0.1	2.1 ± 0.2	2.0 ± 0.1
**3**	3.2 ± 0.2	3.9 ± 0.4	17.7 ± 0.9	16.8 ± 2.6	6.9 ± 1.3	4.6 ± 0.9	3.3 ± 0.1	3.8 ± 0.7
**4**	21.8 ± 0.2	>50	>50	>50	>50	>50	>50	2.9 ± 0.1
**5**	23.6 ± 0.8	21.5 ± 1.6	33.8 ± 2.9	>50	>50	>50	>50	23.8 ± 0.9
**6**	35.6 ± 0.1	>50	>50	>50	>50	32.8 ± 0.9	>50	>50
HL	>50	>50	>50	>50	>50	>50	>50	>50
cisplatin	27.0 ± 5.3	18.2 ± 0.8	23.0 ± 2.6	>50	>50	>50	>50	7.5 ± 0.1

## Data Availability

The Cambridge Crystallographic Database contains the supplementary crystallographic data for the [Cu(phen)(L)(NO_3_)] complex, CCDC deposition number: 2223833. These data can be obtained free of charge via www.ccdc.cam.ac.uk/conts/retrieving.html (or from the Cambridge Crystallographic Data Centre, 12 Union Road, Cambridge CB21EZ, UK; fax: (+44) 1223-336-033; or deposit@ccdc.cam.ac.uk). Supplementary Materials to this article can be found online at https://www.mdpi.com/article/10.3390/pharmaceutics15020307/s1.

## Supplementary Materials

The following supporting information can be downloaded at: https://www.mdpi.com/article/10.3390/pharmaceutics15020307/s1, Figure S1: The dose–response curves of complexes **1**–**6**; Figure S2: The FTIR spectrum of complex **1** measured by the ATR technique; Figure S3: The FTIR spectrum of complex **2** measured by the ATR technique; Figure S4: The FTIR spectrum of complex **3** measured by the ATR technique; Figure S5: The FTIR spectrum of complex **4** measured by the ATR technique; Figure S6: The FTIR spectrum of complex **5** measured by the ATR technique; Figure S7: The FTIR spectrum of complex **6** measured by the ATR technique; Figure S8: The comparison of electronic spectra of complex **1** (here labelled under code of 72) measured in the solid state (nujol; blue dotted line), and in the MeCN (black solid line) and MeNO_2_ (red dashed line) solutions; Figure S9: The comparison of the electronic spectra of complex **2** (here labelled under code of 74) measured in the solid state (nujol; blue dotted line), and in the MeCN (black solid line) and MeNO_2_ (red dashed line) solutions; Figure S10: The comparison of the electronic spectra of complex **3** (here labelled under code of 76) measured in the solid state (nujol; blue dotted line), and in the MeCN (black solid line) and MeNO_2_ (red dashed line) solutions; Figure S11: The comparison of electronic spectra of complex **4** (here labelled under code of 77) measured in the solid state (nujol; blue dotted line), and in the MeCN (black solid line) and MeNO_2_ (red dashed line) solutions; Figure S12: The comparison of the electronic spectra of complex **5** (here labelled under code of 78) measured in the solid state (nujol; blue dotted line), and in the MeCN (black solid line) and MeNO_2_ (red dashed line) solutions; Figure S13: The comparison of electronic spectra of complex **6** (here labelled under code of 79) measured in the solid state (nujol; blue dotted line), and in the MeCN (black solid line) and MeNO_2_ (red dashed line) solutions; Figure S14: ESI-MS spectrum of complex **1** measured in the MeOH solution; Figure S15: ESI-MS spectrum of complex **2** measured in the MeOH solution; Figure S16: ESI-MS spectrum of complex **3** measured in the MeOH solution; Figure S17: ESI-MS spectrum of complex **4** measured in the MeOH solution; Figure S18: ESI-MS spectrum of complex **5** measured in the MeOH solution; Figure S19: ESI-MS spectrum of complex **6** measured in the MeOH solution; Table S1: The results of the conductivity experiments for complexes **1**–**6** in MeCN and MeNO_2_; Figure S20: A part of the crystal structure of [Cu(phen)(L)(NO_3_)] showing the C–H···O, C–H···C (red dashed lines), and C···C (cyan dashed lines) non-covalent contacts; Table S2: The coordinates (XYZ format) for the DFT/ωB97X-D/def2-tzvp optimized geometries of the complex species of [Cu(bphen)(L)(NO_3_)] (2) and [Cu(bphen)(L)]^+^; Table S3: Identification of the coordination polyhedron shape and its deformation in the vicinity of the Cu(II) atom in the X-ray structure of [Cu(phen)(L)(NO_3_)]; Table S4: Selected non-covalent contacts in the crystal structure of [Cu(phen)(L)(NO_3_)]; Figure S21: Supplementary data to Figure 5, showing the representative samples of cell cycle analysis in the A2780 cells treated by the half-cytotoxic concentrations of the tested compounds and the untreated control after 24 h of incubation using the BD Cycletest^TM^ Plus DNA Kit (Becton Dickinson, Franklin Lakes, NJ, USA); Figure S22: Supplementary data to Figure 6, showing the representative samples of the flow cytometry analysis of the A2780 cells treated by the half-cytotoxic concentrations of the tested compounds and untreated control after 24 h of incubation using propidium iodide (PI) and Annexin V-FITC Apoptosis Detection Kits (Enzo Life Sciences, Farmingdale, NY, USA); Figure S23: Supplementary data to Figure 7, showing the representative samples of the flow cytometry analysis of the A2780 cells treated by half-cytotoxic concentrations of the tested compounds, positive control, and untreated control after 24 h of incubation using the CellEvent^TM^ Caspase-3/7 Green Flow Cytometry Assay Kit (Thermo Fisher Scientific, Waltham, MA, USA); Figure S24: Supplementary data to Figure 8, showing the representative samples of the flow cytometry analysis of the A2780 cells treated by the half-cytotoxic concentrations of the tested compounds, positive control, and untreated control after 24 h of incubation using the MITO-ID^®^ Membrane Potential Detection Kit (Enzo Life Sciences, Farmingdale, NY, USA); Figure S25: Supplementary data to Figure 10, showing the representative samples of the flow cytometry analysis of the A2780 cells treated by the half-cytotoxic concentrations of the tested compounds, positive control, and untreated control after 24 h of incubation using the CYTO-ID^®^ Autophagy Detection Kit 2.0 (Enzo Life Sciences, Farmingdale, NY, USA); Figure S26: The time-dependent concentration profile of intracellular copper content in the A2780 cells treated by 3 µM solution of complex **2** after 24 h of incubation and in the untreated control; Figure S27: The time-dependent concentration profile of intracellular copper in the 22Rv1 cells treated by the 3 µM solution of complex **2** after 24 h of incubation and in the untreated control; Figure S28: The data regarding the interaction studies of complex **2** with selected proteins.
