# Supplementary material for: Heteroleptic Copper(II) Complexes Containing 2′-Hydroxy-4-(Dimethylamino)Chalcone Show Strong Antiproliferative Activity
Source: Pharmaceutics. 2023 Jan 17;15(2):307. doi: 10.3390/pharmaceutics15020307 (PMC9967299; doi:10.3390/pharmaceutics15020307)

Supplementary Materials

Heteroleptic copper(II) complexes containing 2'-hydroxy-4-(dimethylamino)chalcone show strong antiproliferative activity

Zdeněk Trávníček [1,*], Tomáš Malina [1], Ján Vančo [1], Marek Šebela [2] and Zdeněk Dvořák [3]

[1] Regional Center of Advanced Technologies and Materials, Czech Advanced Technology and Research Institute (CATRIN), Palacký University, Šlechtitelů 241/27, CZ-783 71 Olomouc, Czech Republic

[2] Department of Biochemistry, Faculty of Science, Palacký University, Šlechtitelů 27, CZ-783 71 Olomouc, Czech Republic

[3] Department of Cell Biology and Genetics, Faculty of Science, Palacký University, Šlechtitelů 27, CZ-783 71 Olomouc, Czech Republic

* Correspondence: zdenek.travnicek@upol.cz; Tel.: +420 585634545

Table of Contents	Page
**Figure S1.** The dose-response curves of the complexes **1–6**	S4
**Figure S2.** The FTIR spectrum of complex **1** measured by the ATR technique.	S5
**Figure S3.** The FTIR spectrum of complex **2** measured by the ATR technique.	S5
**Figure S4.** The FTIR spectrum of complex **3** measured by the ATR technique.	S6
**Figure S5.** The FTIR spectrum of complex **4** measured by the ATR technique.	S6
**Figure S6.** The FTIR spectrum of complex **5** measured by the ATR technique.	S7
**Figure S7.** The FTIR spectrum of complex **6** measured by the ATR technique.	S7
**Figure S8.** The comparison of electronic spectra of complex **1** (here labelled under code of 72) measured in the solid state (nujol; blue dotted line), and in MeCN (black solid line) and $MeNO_2$ (red dashed line) solutions.	S8
**Figure S9.** The comparison of electronic spectra of complex **2** (here labelled under code of 74) measured in the solid state (nujol; blue dotted line), and in MeCN (black solid line) and $MeNO_2$ (red dashed line) solutions.	S8
**Figure S10.** The comparison of electronic spectra of complex **3** (here labelled under code of 76) measured in the solid state (nujol; blue dotted line), and in MeCN (black solid line) and $MeNO_2$ (red dashed line) solutions.	S9
**Figure S11.** The comparison of electronic spectra of complex **4** (here labelled under code of 77) measured in the solid state (nujol; blue dotted line), and in MeCN (black solid line) and $MeNO_2$ (red dashed line) solutions.	S9
**Figure S12.** The comparison of electronic spectra of complex **5** (here labelled under code of 78) measured in the solid state (nujol; blue dotted line), and in MeCN (black solid line) and $MeNO_2$ (red dashed line) solutions.	S10
**Figure S13.** The comparison of electronic spectra of complex **6** (here labelled under code of 79) measured in the solid state (nujol; blue dotted line), and in MeCN (black solid line) and $MeNO_2$ (red dashed line) solutions.	S10
**Figure S14.** ESI-MS spectrum of complex **1** measured in MeOH solution.	S11
**Figure S15.** ESI-MS spectrum of complex **2** measured in MeOH solution.	S11
**Figure S16.** ESI-MS spectrum of complex **3** measured in MeOH solution.	S12
**Figure S17.** ESI-MS spectrum of complex **4** measured in MeOH solution.	S12
**Figure S18.** ESI-MS spectrum of complex **5** measured in MeOH solution.	S13
**Figure S19.** ESI-MS spectrum of complex **6** measured in MeOH solution.	S13
**Table S1** The results of conductivity experiments for complexes **1–6** in MeCN and $MeNO_2$	S14
**Figure S20**. A part of the crystal structure of [Cu(phen)(L)(NO_3)] showing the C–H···O, C–H···C (red dashed lines) and C···C (cyan dashed lines) non-covalent contacts.	S14
**Table S2**. The coordinates (XYZ format) for the DFT/ωB97X-D/def2-tzvp optimized geometries of the complex species of [Cu(bphen)(L)(NO_3)] (**2**) and $[Cu(bphen)(L)]^+$.	S15-S16
**Table S3**. Identification of the coordination polyhedron shape and its deformation in the vicinity of the Cu(II) atom in X-ray structure of [Cu(phen)(L)(NO_3)].	S17

**Table S4**. Selected non-covalent contacts in the crystal structure of [Cu(phen)(L)(NO_3)].	S17
**Figure S21**. Supplementary data to **Figure 5**, showing the representative samples of cell cycle analysis in A2780 cells treated by half-cytotoxic concentrations of the tested compounds and untreated control after 24 h of incubation using BD Cycletest^{TM} Plus DNA kit (Becton Dickinson, USA).	S18
**Figure S22**. Supplementary data to **Figure 6**, showing the representative samples of flow cytometry analysis of A2780 cells treated by half-cytotoxic concentrations of the tested compounds and untreated control after 24 h of incubation using propidium iodide (PI) and Annexin V-FITC apoptosis detection kits (Enzo Life Sciences, USA).	S19
**Figure S23**. Supplementary data to **Figure 7**, showing the representative samples of flow cytometry analysis of A2780 cells treated by half-cytotoxic concentrations of the tested compounds, positive control, and untreated control after 24 h of incubation using CellEvent^{TM} Caspase-3/7 Green Flow Cytometry Assay Kit (Thermo Fisher Scientific, USA).	S20
**Figure S24**. Supplementary data to **Figure 8**, showing the representative samples of flow cytometry analysis of A2780 cells treated by half-cytotoxic concentrations of the tested compounds, positive control, and untreated control after 24 h of incubation using MITO-ID® Membrane potential detection kit (Enzo Life Sciences, USA).	S21
**Figure S25**. Supplementary data to **Figure 10**, showing the representative samples of flow cytometry analysis of A2780 cells treated by half-cytotoxic concentrations of the tested compounds, positive control, and untreated control after 24 h of incubation using CYTO-ID® Autophagy Detection Kit 2.0 (Enzo Life Sciences, USA).	S22
**Figure S26**. The time-dependent concentration profile of intracellular copper content in the A2780 cells treated by 3 μM solution of complex **2** after 24 h incubation and in untreated control.	S23
**Figure S27**. The time-dependent concentration profile of intracellular copper in the 22Rv1 cells treated by 3 μM solution of complex **2** after 24 h incubation and in untreated control.	S23
**Figure S28.** The data regarding the interaction studies of complex **2** with selected proteins.	S24-S26

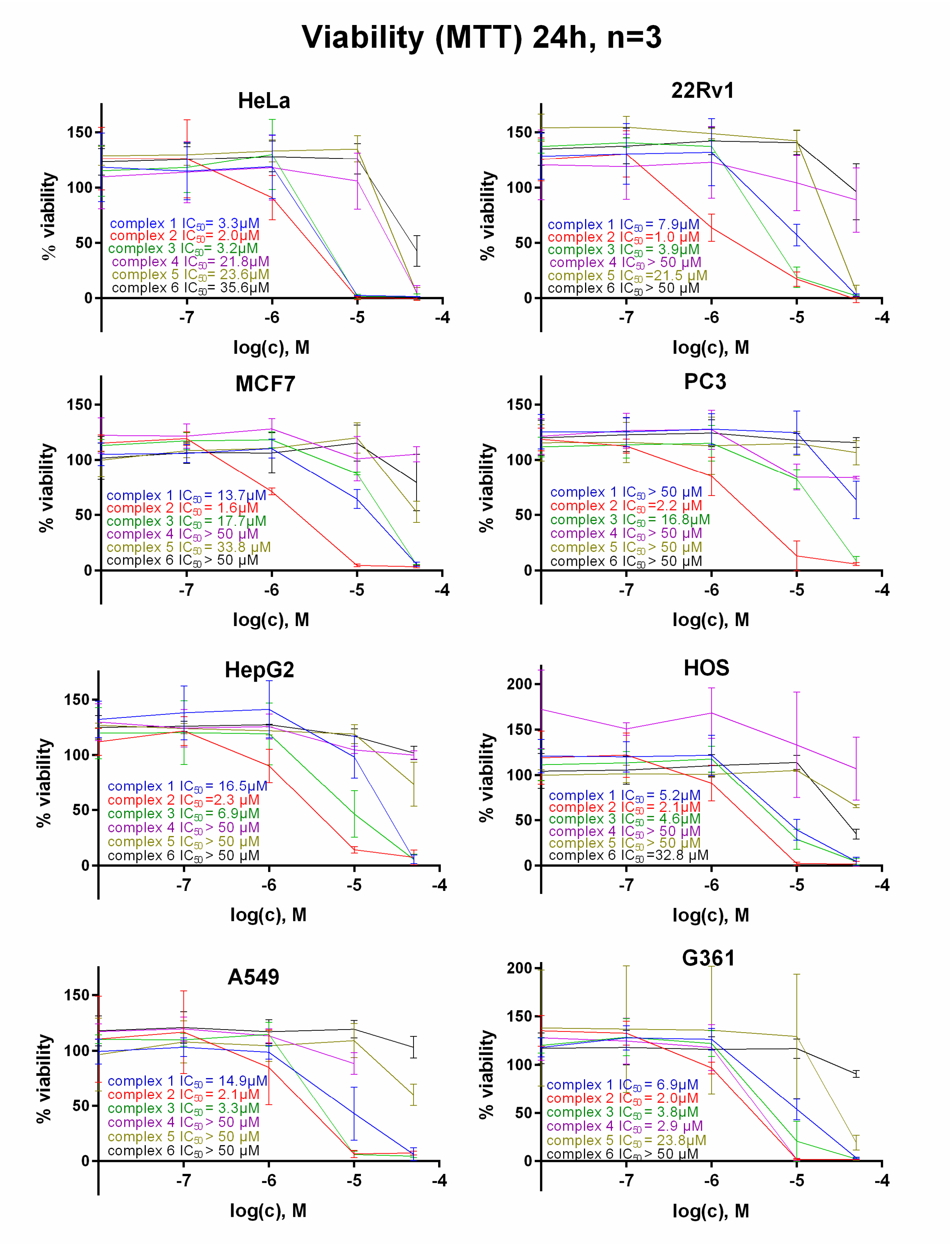

Figure S1 The dose-response curves for complexes **1**–**6**.

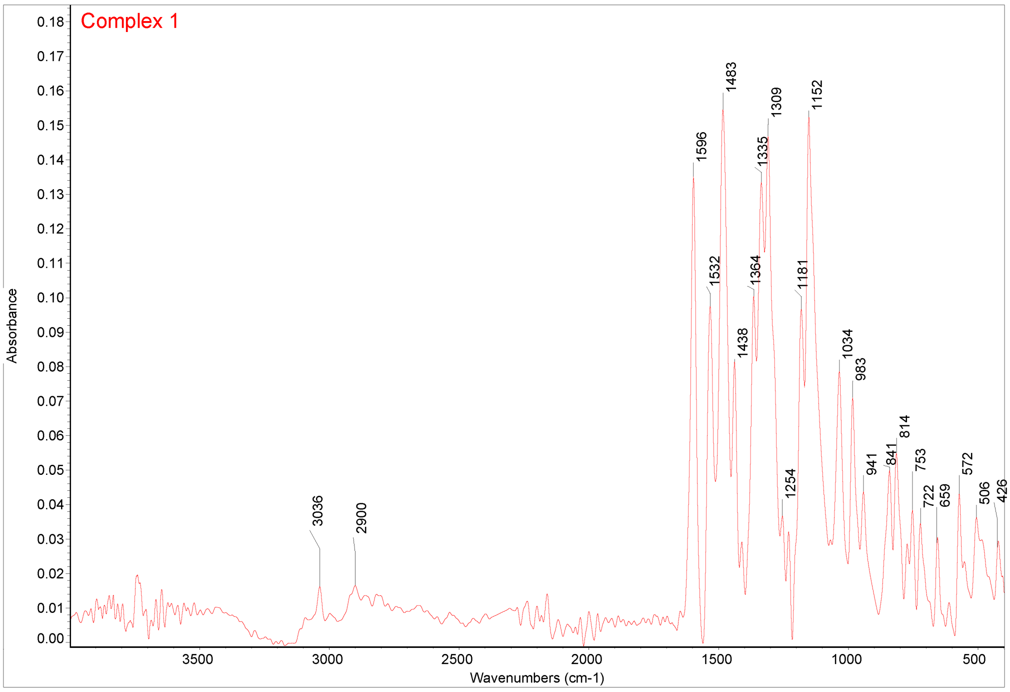

Figure S2 The FTIR spectrum of complex **1** measured by the ATR technique.

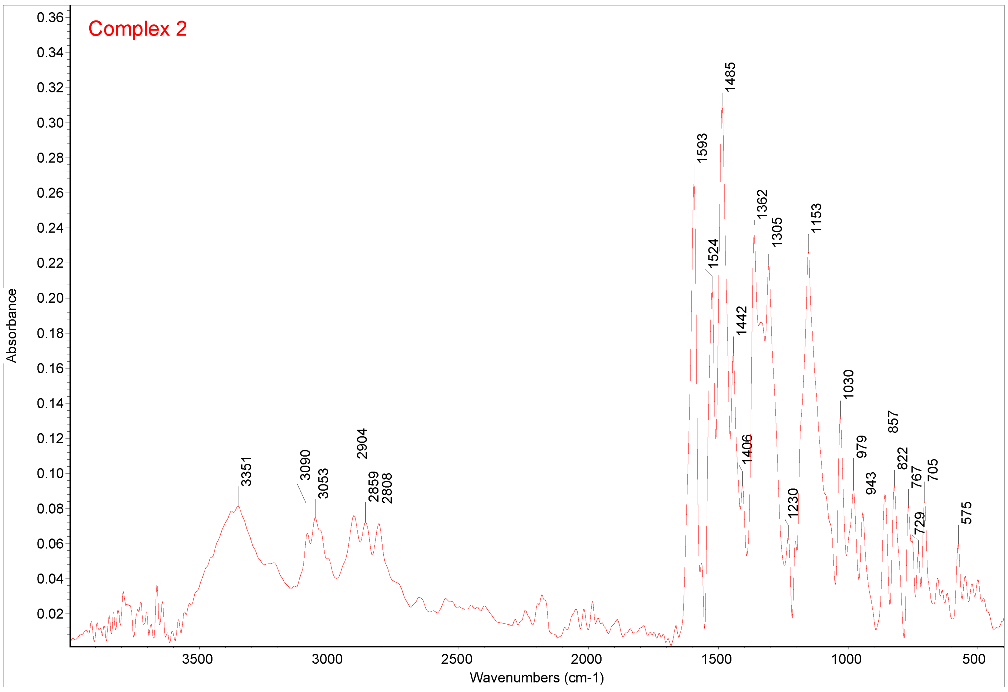

Figure S3 The FTIR spectrum of complex **2** measured by the ATR technique.

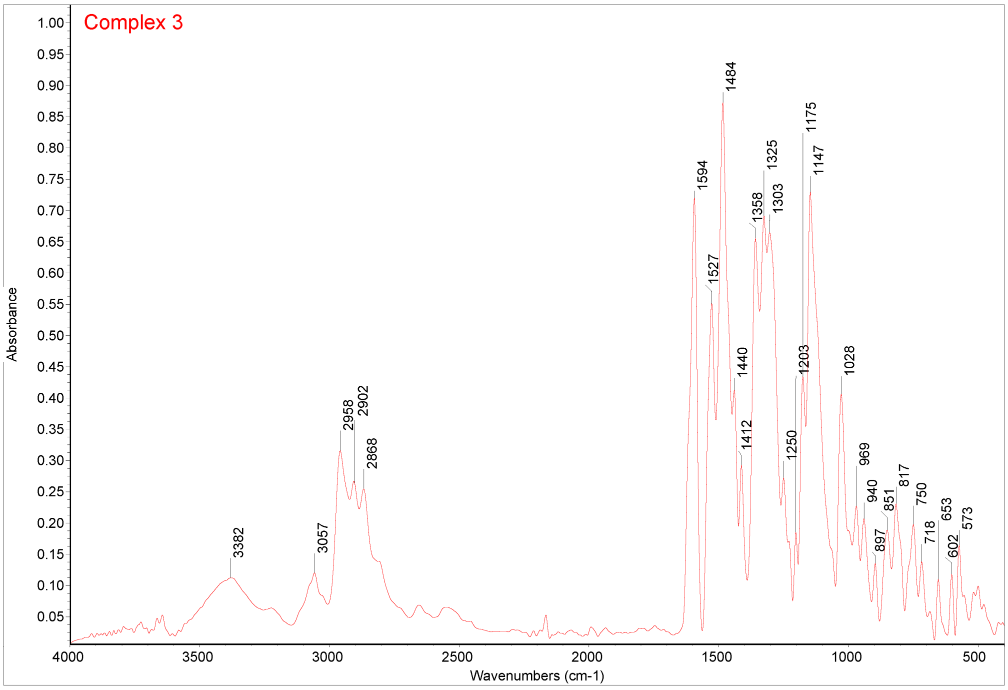

Figure S4 The FTIR spectrum of complex **3** measured by the ATR technique.

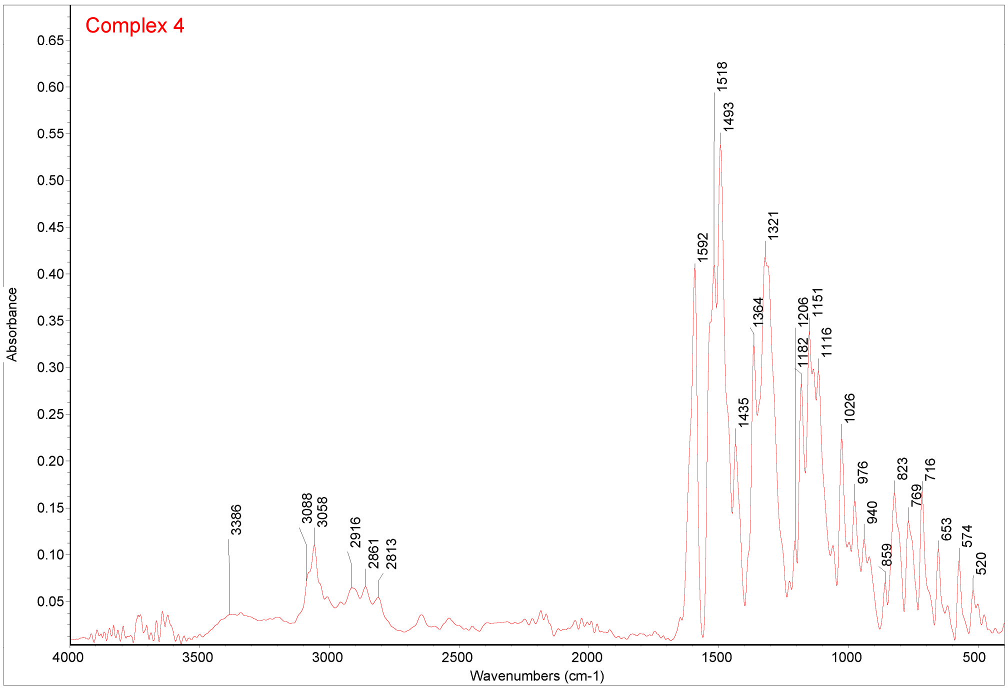

Figure S5 The FTIR spectrum of complex **4** measured by the ATR technique.

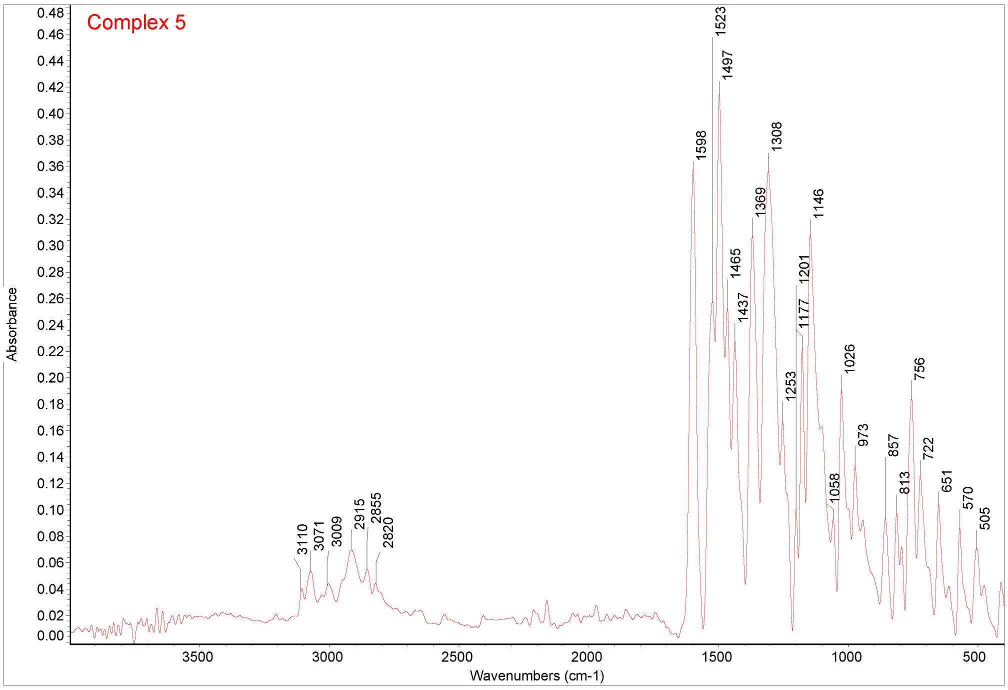

Figure S6 The FTIR spectrum of complex **5** measured by the ATR technique.

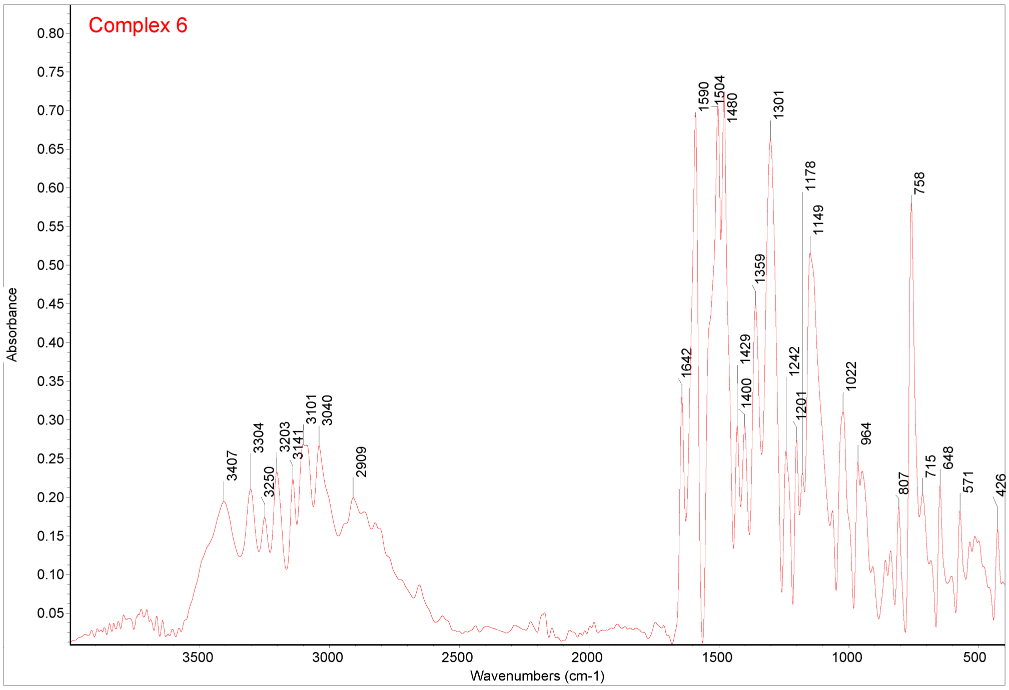

Figure S7 The FTIR spectrum of complex **6** measured by the ATR technique.

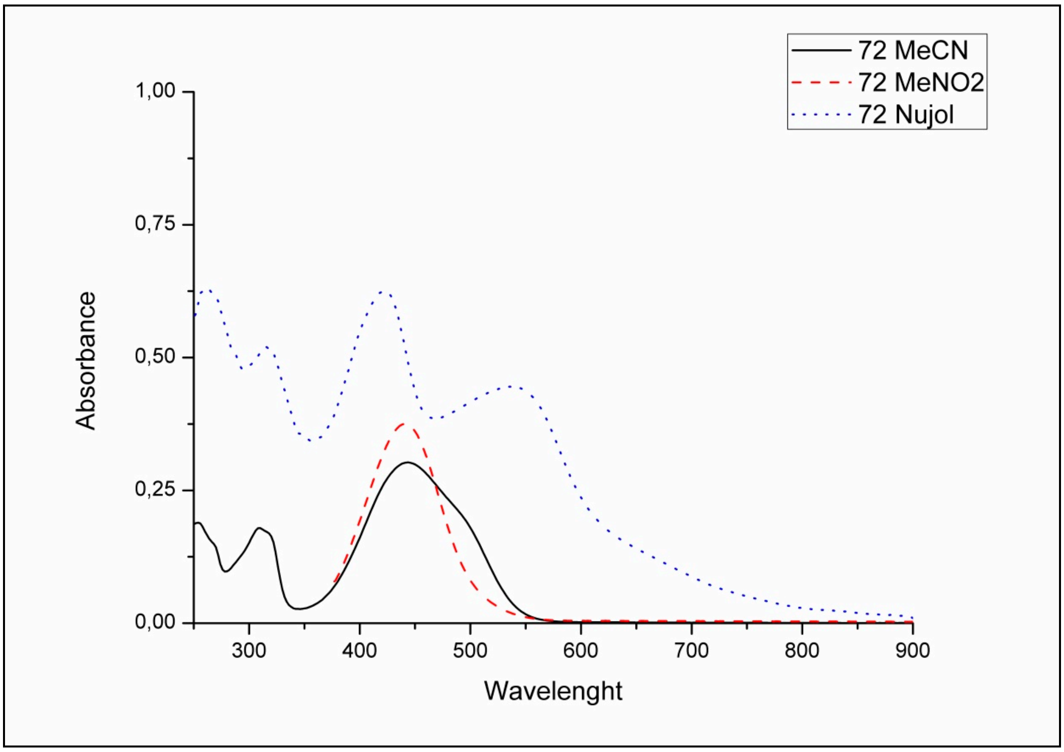

Figure S8 The comparison of electronic spectra of complex **1** (here labelled under code of 72) measured in the solid state (nujol; blue dotted line), and in MeCN (black solid line) and $MeNO_2$ (red dashed line) solutions.

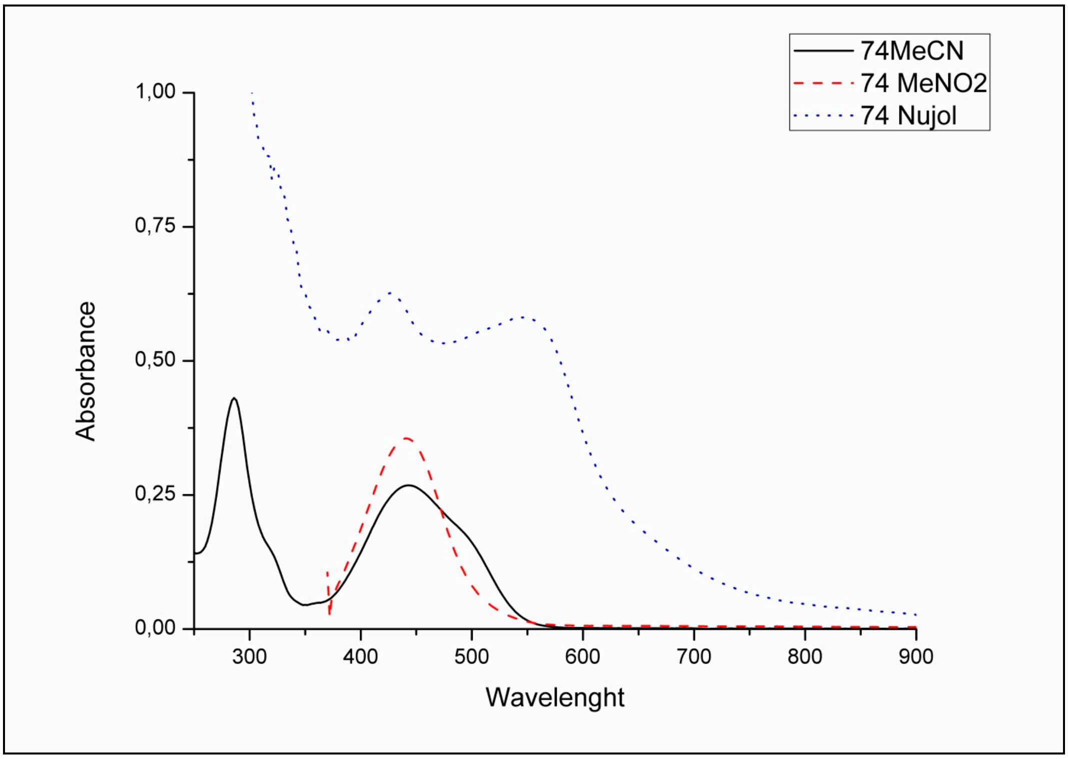

Figure S9 The comparison of electronic spectra of complex **2** (here labelled under code of 74) measured in the solid state (nujol; blue dotted line), and in MeCN (black solid line) and $MeNO_2$ (red dashed line) solutions.

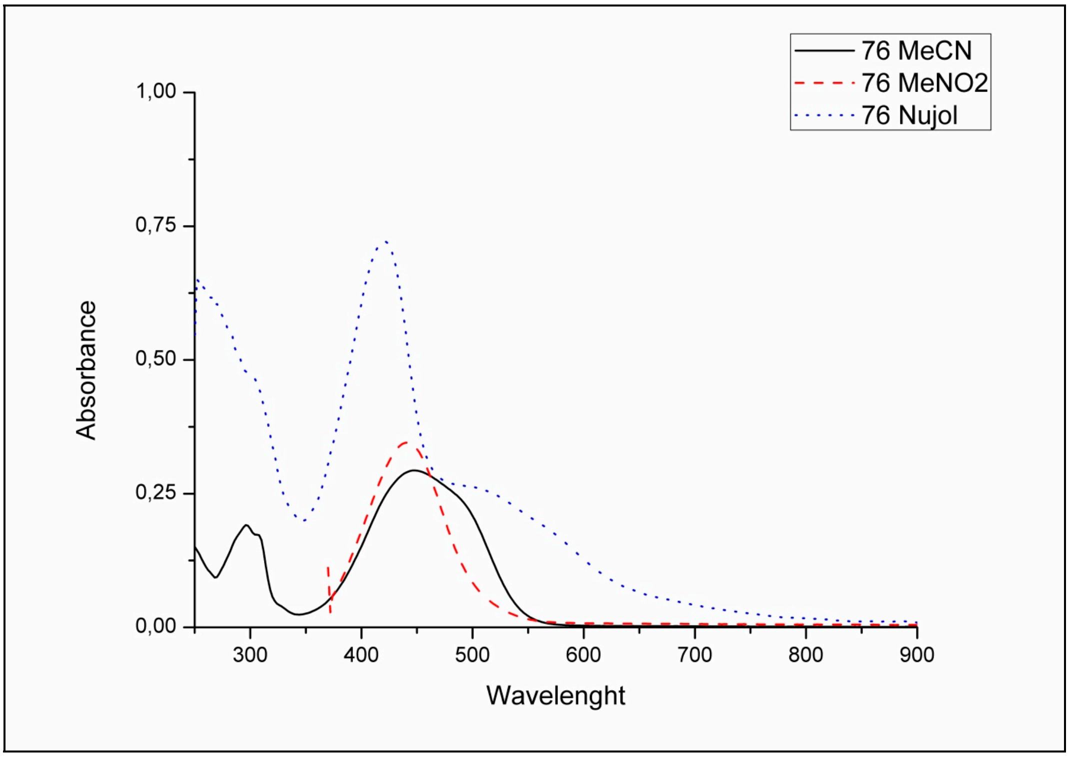

Figure S10 The comparison of electronic spectra of complex **3** (here labelled under code of 76) measured in the solid state (nujol; blue dotted line), and in MeCN (black solid line) and $MeNO_2$ (red dashed line) solutions.

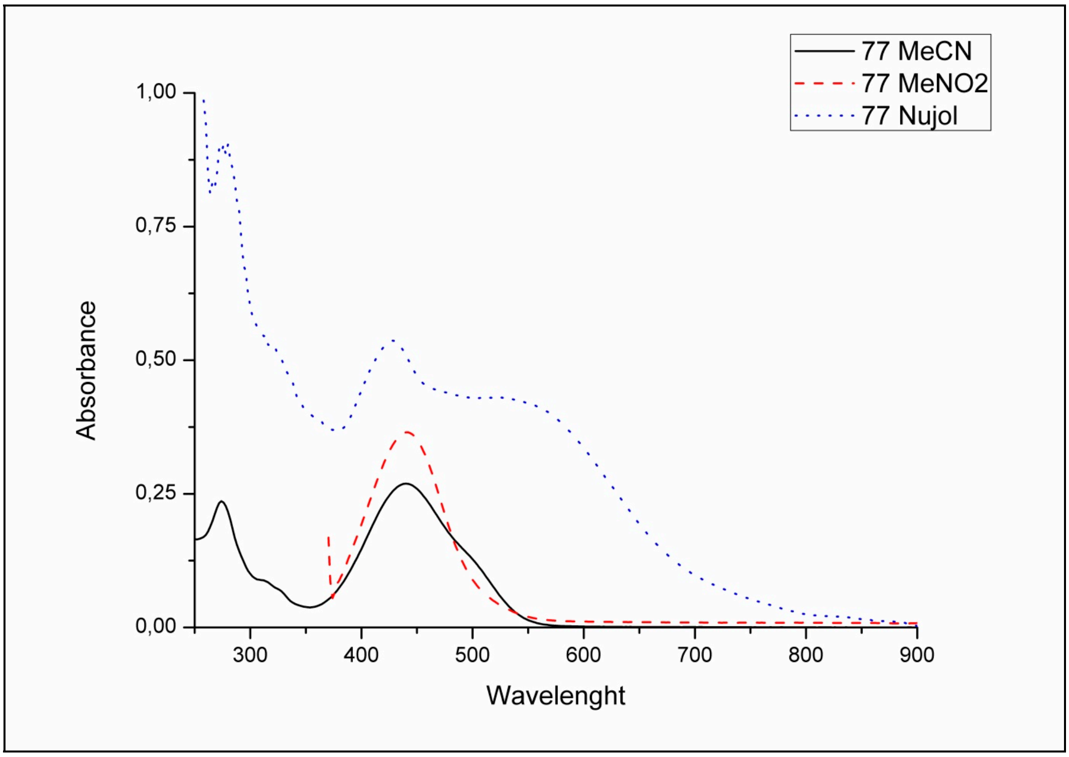

Figure S11 The comparison of electronic spectra of complex **4** (here labelled under code of 77) measured in the solid state (nujol; blue dotted line), and in MeCN (black solid line) and $MeNO_2$ (red dashed line) solutions.

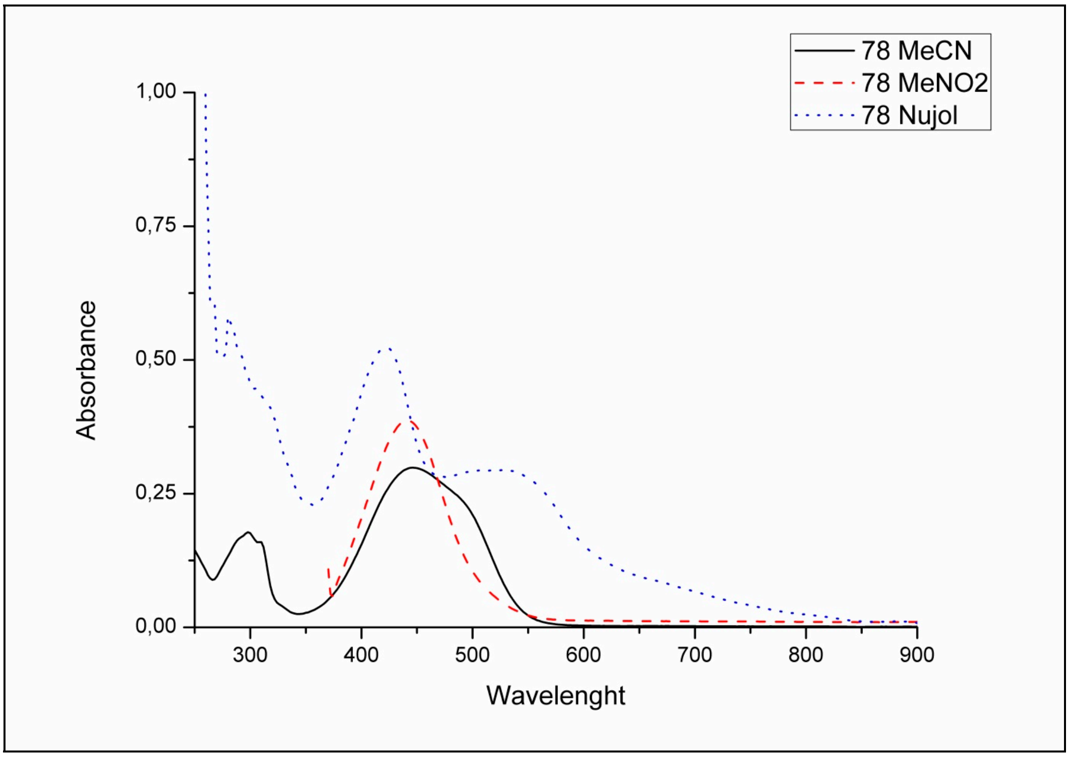

Figure S12 The comparison of electronic spectra of complex **5** (here labelled under code of 78) measured in the solid state (nujol; blue dotted line), and in MeCN (black solid line) and $MeNO_2$ (red dashed line) solutions.

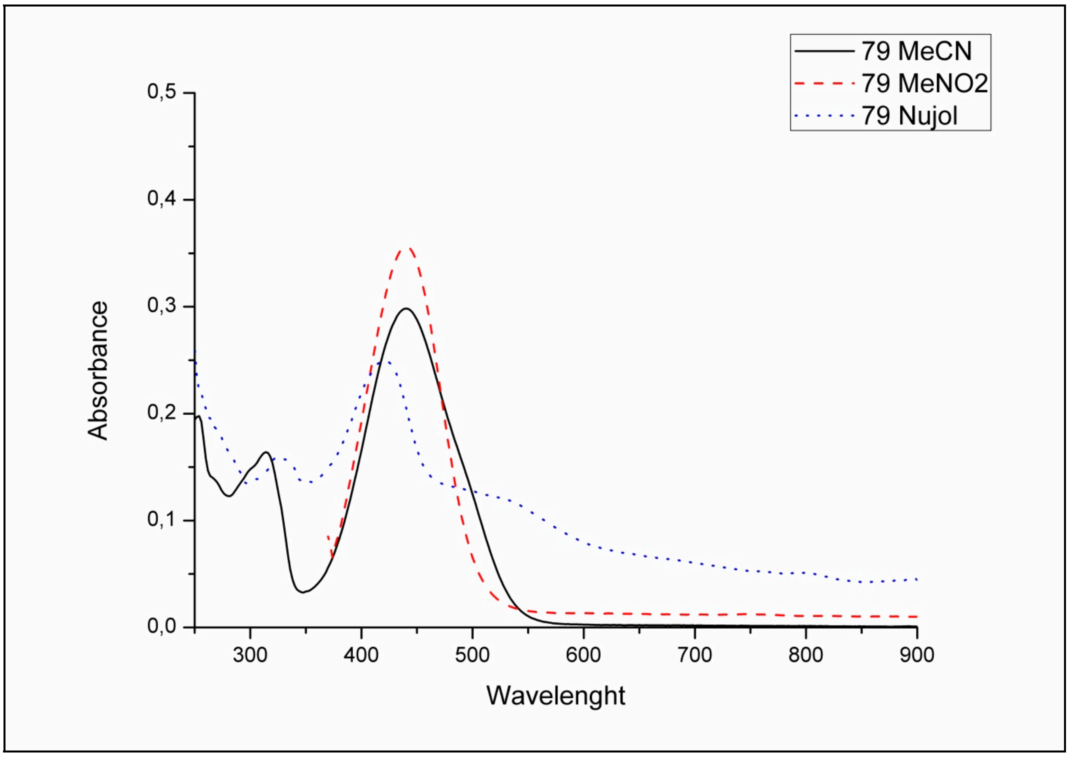

Figure S13 The comparison of electronic spectra of complex **6** (here labelled under code of 79) measured in the solid state (nujol; blue dotted line), and in MeCN (black solid line) and $MeNO_2$ (red dashed line) solutions.

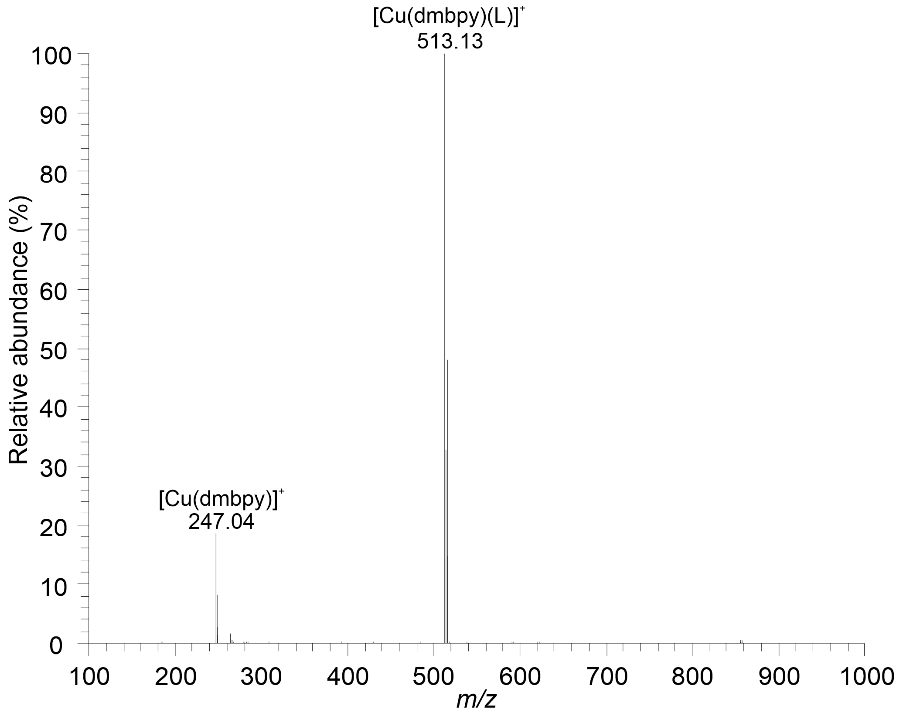

Figure S14. ESI-MS spectrum of complex **1** measured in MeOH solution.

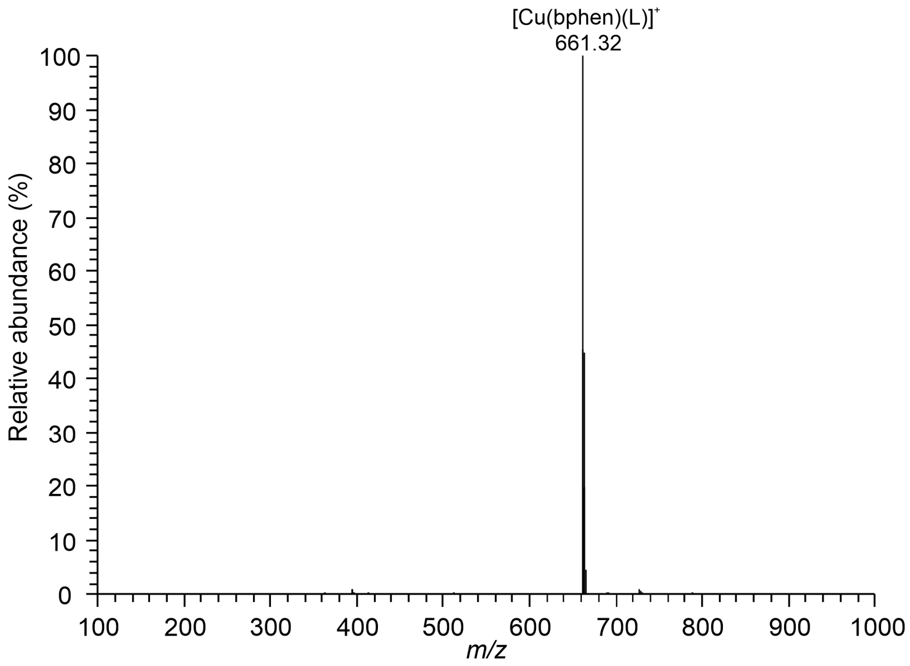

Figure S15. ESI-MS spectrum of complex **2** measured in MeOH solution.

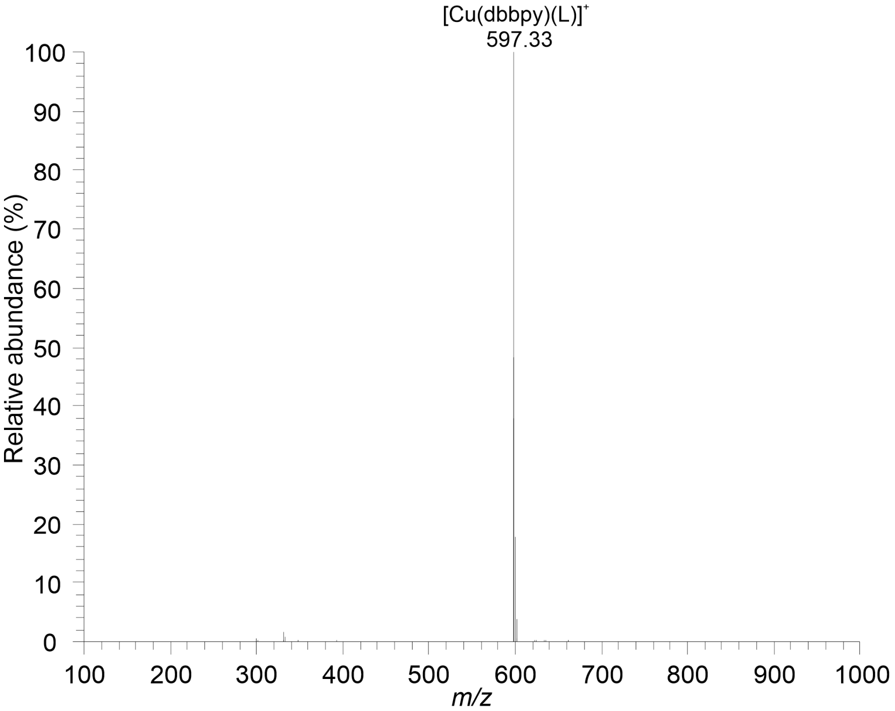

Figure S16. ESI-MS spectrum of complex **3** measured in MeOH solution.

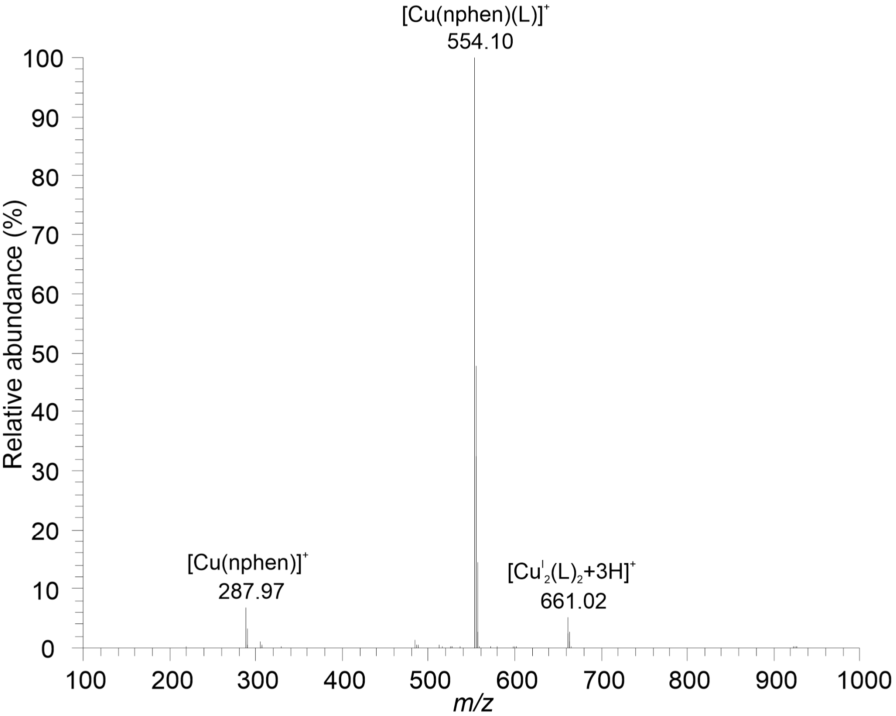

Figure S17. ESI-MS spectrum of complex **4** measured in MeOH solution.

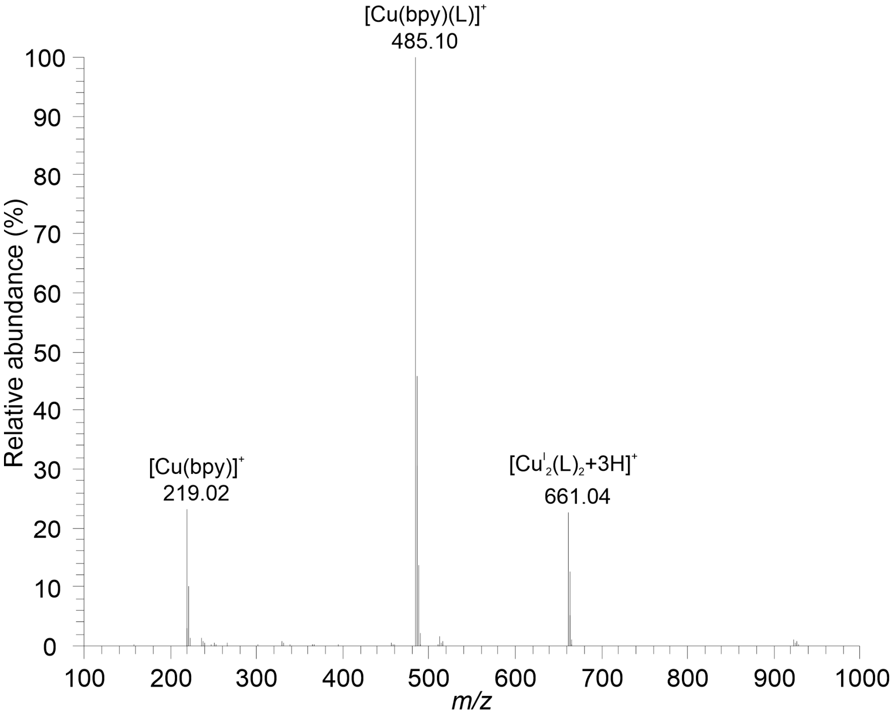

Figure S18. ESI-MS spectrum of complex **5** measured in MeOH solution.

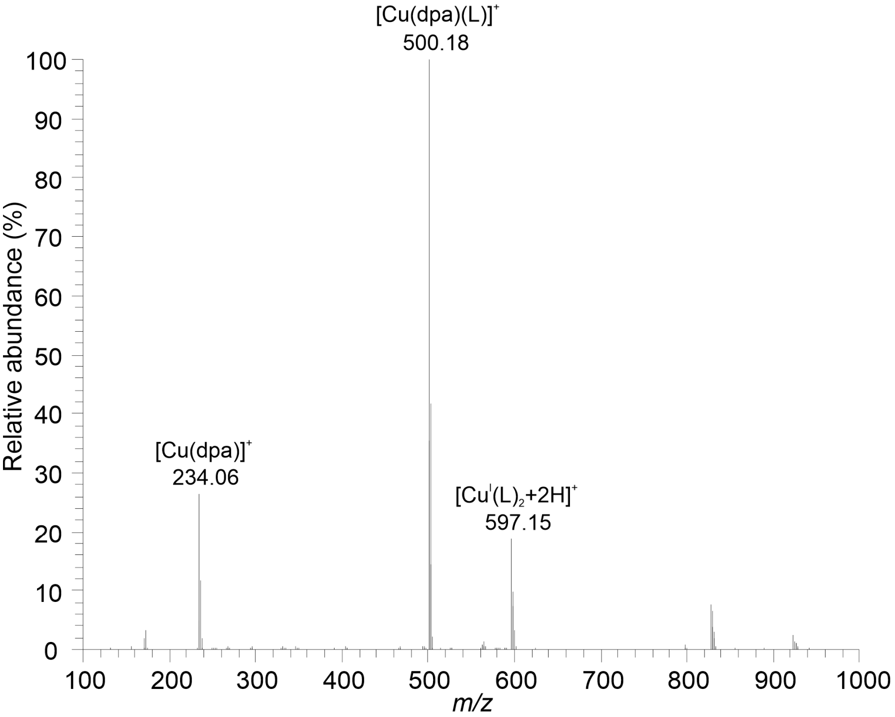

Figure S19. ESI-MS spectrum of complex **6** measured in MeOH solution.

Table S1 The results of conductivity experiments for complexes **1–6** in MeCN and $MeNO_2$.

**Complex**	**Molar conductivity Λ_m (at 25°C, S cm^2 mol^{-1})**	
	**MeCN**	**$MeNO_2$**
**1**	132	76
**2**	133	77
**3**	141	77
**4**	123	75
**5**	140	77
**6**	123	77
1:1 electrolyte*	120-160	75-95

* The data adopted from ref. [19]: W.J. Geary. The use of conductivity measurements in organic solvents for the characterisation of coordination compounds. *Coord. Chem. Rev.* 7 (1971) 81–122.

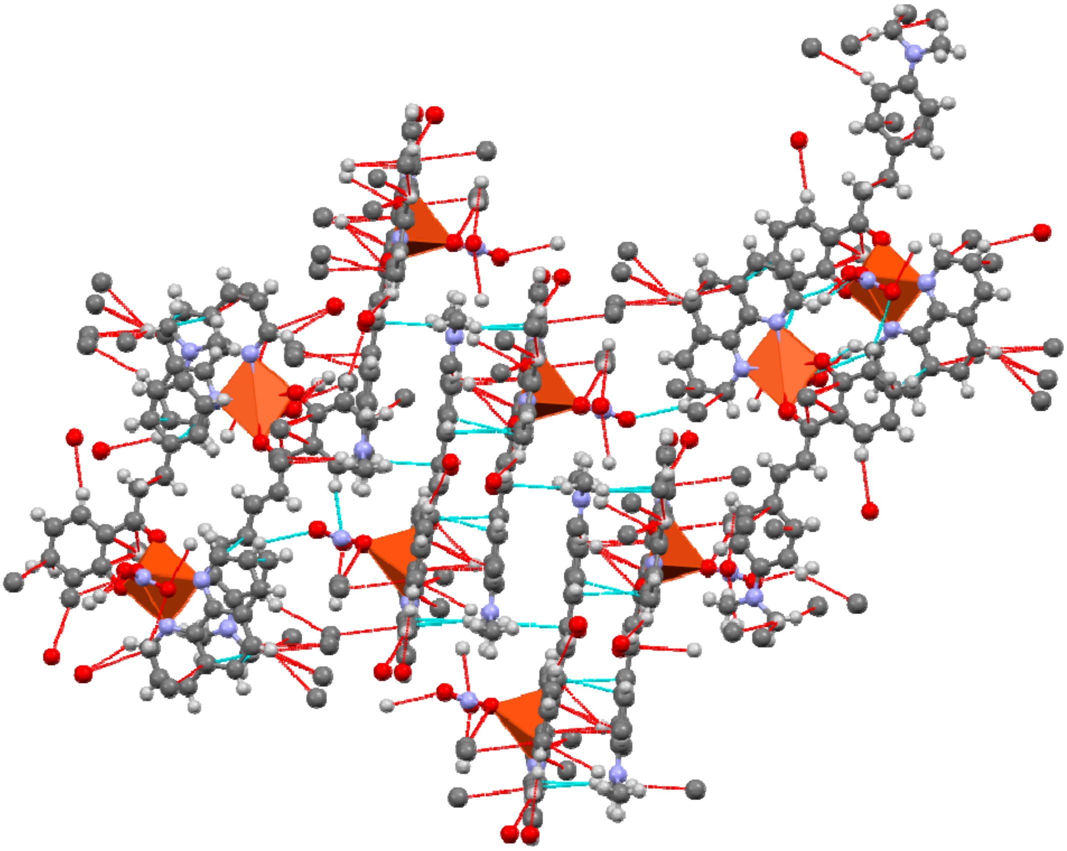

Figure S20. A part of the crystal structure of [Cu(phen)(L)(NO_3)] showing the C–H···O, C–H···C (red dashed lines) and C···C (cyan dashed lines) non-covalent contacts.

Table S2. The coordinates (XYZ format) for the DFT/ωB97X-D/def2-tzvp optimized geometries of the complex species of [Cu(bphen)(L)(NO_3)] (**2**) and $[Cu(bphen)(L)]^+$.

For [Cu(bphen)(L)(NO_3)] (**2**)

Cu	1.266880	0.383530	-1.304381	H	-0.294893	-4.789612	5.492875
N	-0.461648	0.828315	-0.348473	C	1.712650	-5.583166	5.282345
C	-1.015790	0.202873	0.667852	C	2.873934	-5.511734	4.475289
H	-0.462956	-0.628741	1.083478	H	3.738049	-6.114669	4.713188
C	-2.258436	0.573879	1.178547	C	2.928104	-4.680712	3.383622
H	-2.676784	0.010977	2.001249	H	3.841411	-4.659998	2.802103
C	-2.958983	1.619761	0.618176	N	1.658761	-6.397479	6.367143
C	-2.355064	2.323226	-0.454956	C	2.784102	-7.245727	6.702244
C	-1.107621	1.876656	-0.896871	H	3.015457	-7.948741	5.895871
C	-0.446776	2.533821	-1.986924	H	2.541103	-7.819687	7.591754
N	0.733351	2.010895	-2.371705	H	3.681637	-6.657064	6.911652
C	1.369780	2.549962	-3.388903	C	0.447492	-6.477519	7.157832
H	2.310450	2.091086	-3.665508	H	-0.393573	-6.858132	6.570295
C	0.870796	3.662215	-4.065329	H	0.168173	-5.501066	7.562256
H	1.441304	4.078894	-4.883510	H	0.612828	-7.152108	7.993114
C	-0.318812	4.241027	-3.676965	O	0.424418	-1.227125	-2.805853
C	-1.026153	3.647432	-2.600071	N	-0.465314	-1.022194	-3.671135
C	-2.300683	4.093848	-2.119028	O	-0.419936	-1.643467	-4.740444
H	-2.769148	4.947431	-2.587971	O	-1.373179	-0.212271	-3.444372
C	-2.929547	3.466725	-1.100418	C	-4.300160	1.967628	1.141553
H	-3.886631	3.833950	-0.757728	C	-6.829118	2.538722	2.162019
O	3.014018	0.333100	-2.057080	C	-4.468591	2.233621	2.498311
C	3.758473	-0.725927	-2.095910	C	-5.411344	1.985168	0.300776
C	4.771503	-0.776836	-3.086562	C	-6.669196	2.264582	0.810749
H	4.856811	0.074932	-3.751217	C	-5.726363	2.524369	3.003878
C	5.594326	-1.863389	-3.224873	H	-3.608783	2.222410	3.157158
H	6.344088	-1.868909	-4.007791	H	-5.293509	1.761005	-0.752431
C	5.465650	-2.969714	-2.374015	H	-7.527209	2.265657	0.150347
H	6.100859	-3.836810	-2.495231	H	-5.844236	2.738073	4.058726
C	4.516851	-2.936867	-1.385756	H	-7.811960	2.760254	2.558481
H	4.416428	-3.800434	-0.743635	C	-0.812679	5.443402	-4.388043
C	3.652561	-1.834781	-1.201911	C	-1.655352	7.715844	-5.762864
C	2.690105	-1.842472	-0.107958	C	-1.068879	6.627654	-3.700041
O	1.732383	-1.032264	-0.048676	C	-0.977382	5.409804	-5.770702
C	2.834131	-2.799958	0.980083	C	-1.403806	6.538846	-6.453030
H	3.753571	-3.360144	1.050483	C	-1.483558	7.758446	-4.385975
C	1.851234	-2.979096	1.883080	H	-0.928612	6.668149	-2.626591
H	0.943835	-2.398069	1.748843	H	-0.778260	4.492302	-6.311099
C	1.843110	-3.869822	3.018898	H	-1.537971	6.497952	-7.526674
C	0.694936	-3.944275	3.817372	H	-1.669712	8.676326	-3.842718
H	-0.162863	-3.331061	3.562901	H	-1.981394	8.599241	-6.297439
C	0.617313	-4.771948	4.914227				

For $[Cu(bphen)(L)]^+$

Cu	1.332055	0.432119	-1.392309	H	3.750203	-4.762658	2.563070
N	-0.421155	0.821729	-0.479696	N	1.476165	-6.563427	6.038560
C	-0.945582	0.222831	0.569524	C	2.595472	-7.413228	6.391326
H	-0.368492	-0.580085	1.007732	H	2.868334	-8.077714	5.566845
C	-2.186779	0.588180	1.086540	H	2.318489	-8.027944	7.242976
H	-2.578825	0.048093	1.936804	H	3.477743	-6.826672	6.665214
C	-2.917842	1.599868	0.501554	C	0.275640	-6.596025	6.848841
C	-2.347171	2.275521	-0.607813	H	-0.600727	-6.873924	6.256334
C	-1.098165	1.838293	-1.053869	H	0.079622	-5.626273	7.316079
C	-0.463063	2.474877	-2.170366	H	0.399409	-7.334040	7.636218
N	0.727749	1.967582	-2.546748	C	-4.254056	1.945915	1.038220
C	1.349187	2.494962	-3.580548	C	-6.769110	2.532360	2.083888
H	2.299551	2.051986	-3.847768	C	-4.399117	2.263378	2.386708
C	0.821506	3.576704	-4.283523	C	-5.381289	1.917743	0.219103
H	1.381099	3.985689	-5.113385	C	-6.632299	2.204951	0.741891
C	-0.379534	4.138667	-3.905646	C	-5.650359	2.561569	2.904418
C	-1.070341	3.557305	-2.810496	H	-3.526862	2.287721	3.028721
C	-2.349394	3.989956	-2.330770	H	-5.281563	1.652531	-0.826368
H	-2.841612	4.816981	-2.822422	H	-7.502516	2.171345	0.098542
C	-2.952741	3.384261	-1.283913	H	-5.750322	2.816735	3.951882
H	-3.912468	3.744482	-0.941841	H	-7.746360	2.761461	2.489790
O	2.979775	0.290210	-2.288216	C	-0.901854	5.313072	-4.641402
C	3.809555	-0.706008	-2.237842	C	-1.808693	7.534945	-6.056428
C	4.870808	-0.700472	-3.176915	C	-1.195991	6.500375	-3.973941
H	4.918321	0.135836	-3.863308	C	-1.060269	5.250766	-6.023927
C	5.790093	-1.713300	-3.234496	C	-1.518372	6.355056	-6.726416
H	6.579997	-1.676771	-3.975813	C	-1.642281	7.606369	-4.680227
C	5.709469	-2.798497	-2.351578	H	-1.058257	6.564352	-2.901547
H	6.422238	-3.610126	-2.408782	H	-0.833977	4.330395	-6.548567
C	4.710737	-2.818304	-1.414963	H	-1.647095	6.292705	-7.799707
H	4.655078	-3.667806	-0.750303	H	-1.858682	8.526900	-4.153150
C	3.746455	-1.788760	-1.309646	H	-2.161796	8.398448	-6.606092
C	2.725117	-1.850607	-0.274175				
O	1.740281	-1.064965	-0.250971				
C	2.822508	-2.830137	0.794647				
H	3.733906	-3.397945	0.890910				
C	1.808084	-3.023762	1.661129				
H	0.908950	-2.433900	1.514142				
C	1.763529	-3.941024	2.772699				
C	0.603321	-4.011195	3.554964				
H	-0.238839	-3.375358	3.304156				
C	0.495559	-4.863442	4.630221				
H	-0.423659	-4.876320	5.197311				
C	1.567051	-5.711681	4.987044				
C	2.742753	-5.639584	4.200037				
H	3.593398	-6.261922	4.435915				
C	2.828300	-4.781347	3.131934				

Table S3. Identification of the coordination polyhedron shape and its deformation in the vicinity of the Cu(II) atom in X-ray structure of [Cu(phen)(L)(NO_3)].

S H A P E v.2.1, Continuous Shape Measures calculation (c) 2013, Electronic Structure Group, Universitat de Barcelona

JTBPY-5	5 D3h	Johnson trigonal bipyramid J12
**SPY-5**	**4 C4v**	**Spherical square pyramid**
TBPY-5	3 D3h	Trigonal bipyramid
vOC-5	2 C4v	Vacant octahedron
PP-5	1 D5h	Pentagon

Structure [ML5]	JTBPY-5	**SPY-5**	TBPY-5	vOC-5	PP-5
Cu	8.829	**1.041**	5.668	1.807	28.505

Table S4. Selected non-covalent contacts in the crystal structure of [Cu(phen)(L)(NO_3)].

D–H···A	d(D–H)	d(H···A)	d(D···A)	<(DHA)
C(2)-H(2A)...O(1)	0.93	2.47	2.941(4)	111.6
C(2)-H(2A)...O(6)#1	0.93	2.46	3.146(5)	130.4
C(10)-H(10A)...O(5)#2	0.93	2.59	3.315(5)	135.3

Symmetry transformations used to generate equivalent atoms: #1 -x+1, -y+1, -z;
#2 x+1/2, -y+3/2, z+1/2.

Complex 2

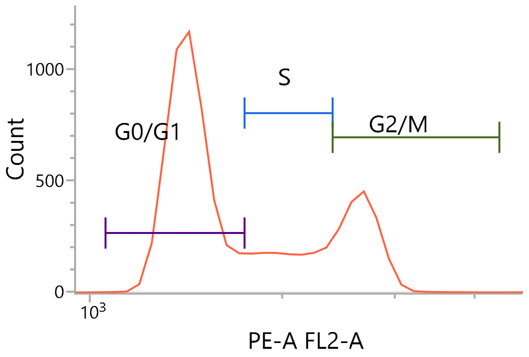

Cisplatin

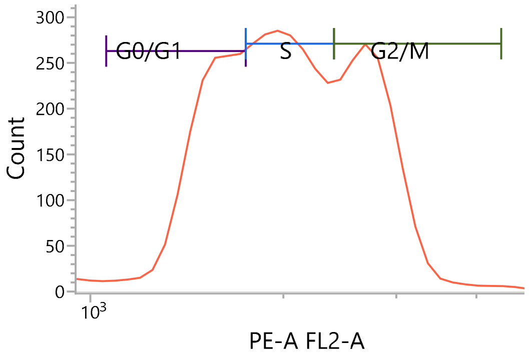

Untreated cells

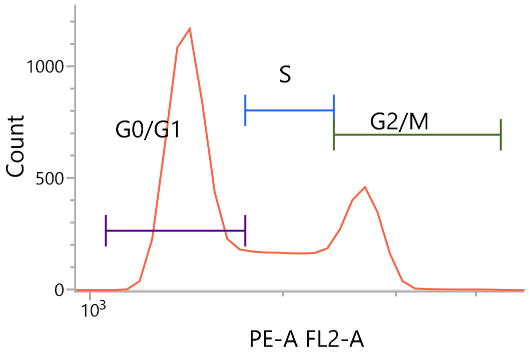

Figure S21. Supplementary data to **Figure 5**, showing the representative samples of cell cycle analysis in A2780 cells treated by half-cytotoxic concentrations of the tested compounds and untreated control after 24 h of incubation using BD Cycletest^{TM} Plus DNA kit (Becton Dickinson, USA).

Complex 2

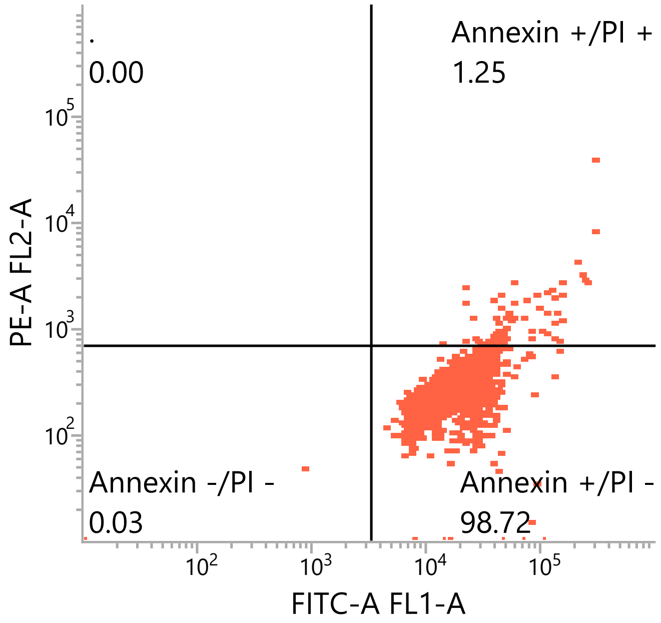

Cisplatin

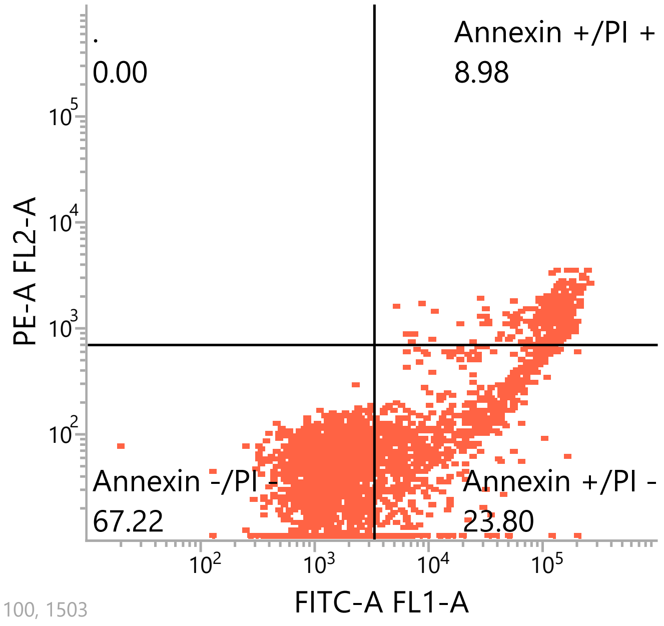

Untreated cells

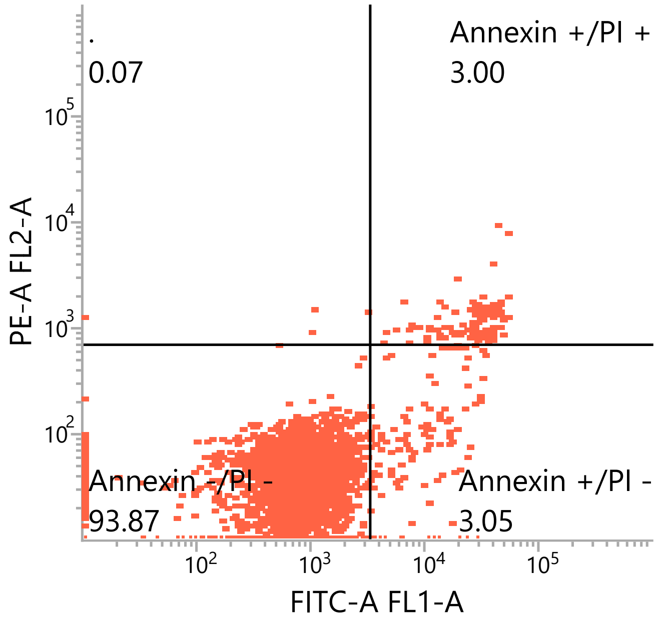

Figure S22. Supplementary data to **Figure 6**, showing the representative samples of flow cytometry analysis of A2780 cells treated by half-cytotoxic concentrations of the tested compounds and untreated control after 24 h of incubation using propidium iodide (PI) and Annexin V-FITC apoptosis detection kits (Enzo Life Sciences, USA).

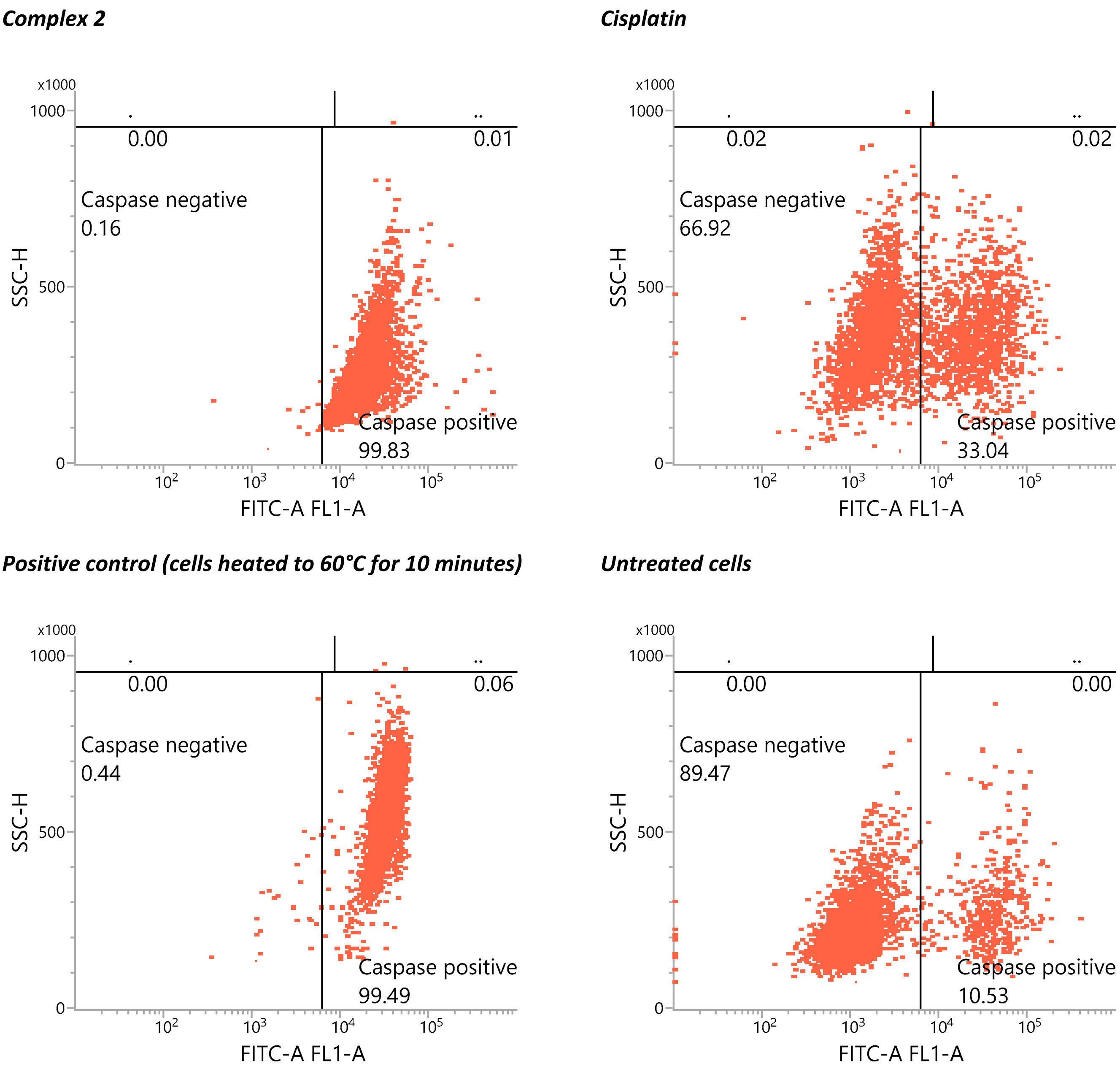

Figure S23. Supplementary data to **Figure 7**, showing the representative samples of flow cytometry analysis of A2780 cells treated by half-cytotoxic concentrations of the tested compounds, positive control, and untreated control after 24 h of incubation using CellEvent™ Caspase-3/7 Green Flow Cytometry Assay Kit (Thermo Fisher Scientific, USA).

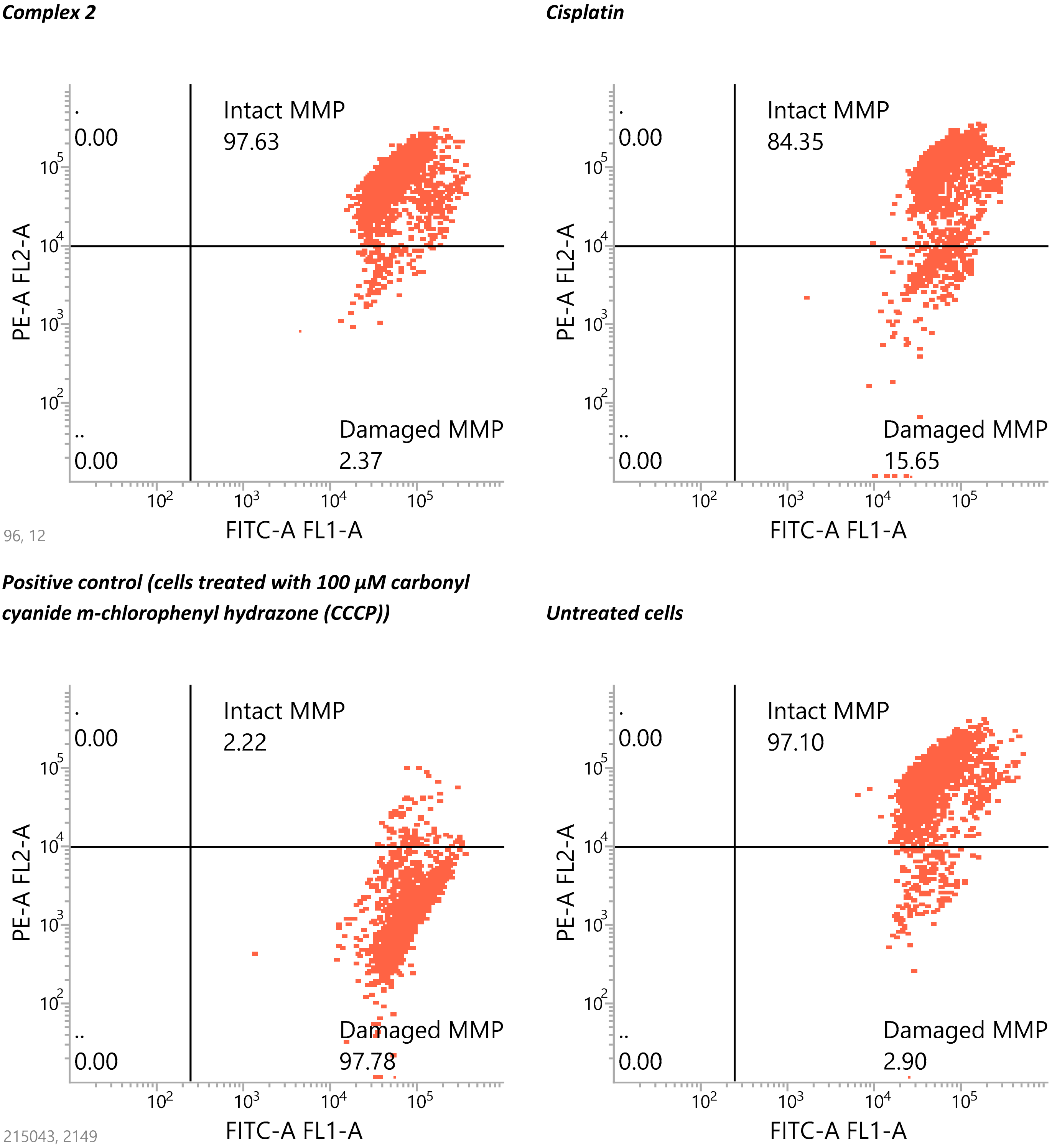

Figure S24. Supplementary data to **Figure 8**, showing the representative samples of flow cytometry analysis of A2780 cells treated by half-cytotoxic concentrations of the tested compounds, positive control, and untreated control after 24 h of incubation using MITO-ID® Membrane potential detection kit (Enzo Life Sciences, USA).

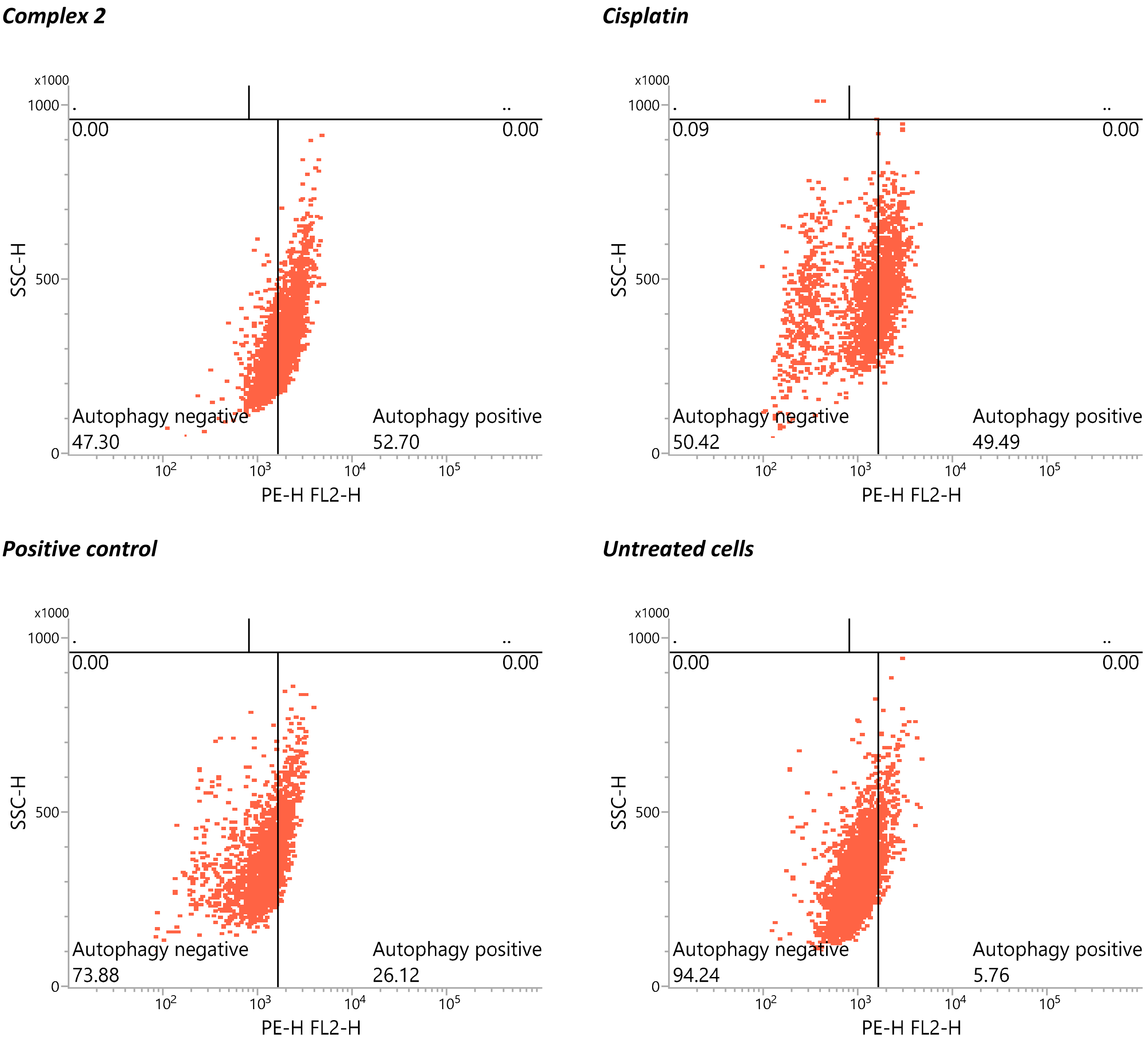

Figure S25. Supplementary data to **Figure 10**, showing the representative samples of flow cytometry analysis of A2780 cells treated by half-cytotoxic concentrations of the tested compounds, positive control, and untreated control after 24 h of incubation using CYTO-ID® Autophagy Detection Kit 2.0 (Enzo Life Sciences, USA).

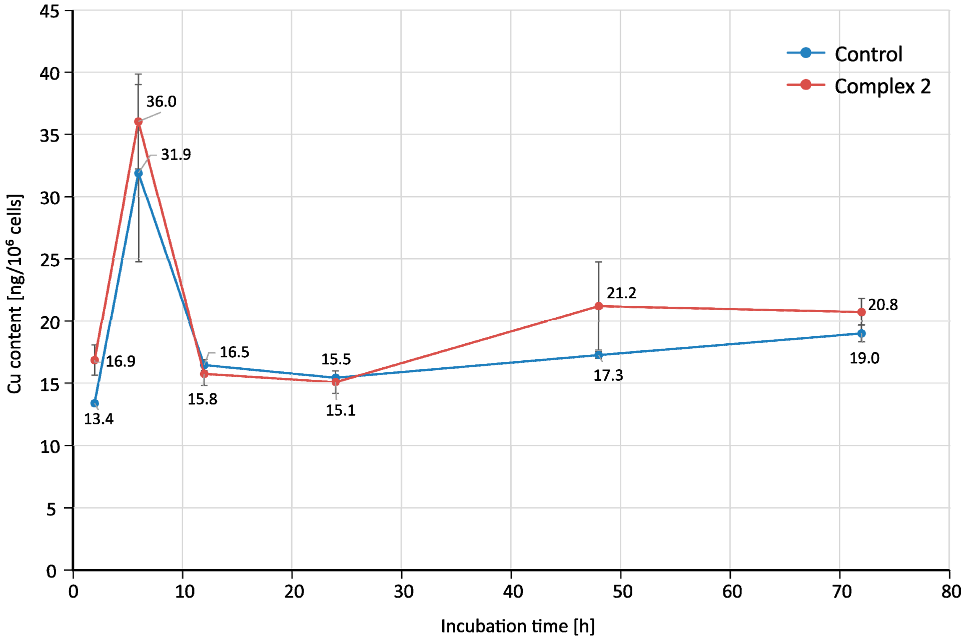

Figure S26. The time-dependent concentration profile of intracellular copper content in the A2780 cells treated by 3 µM solution of complex **2** after 24 h incubation and in untreated control.

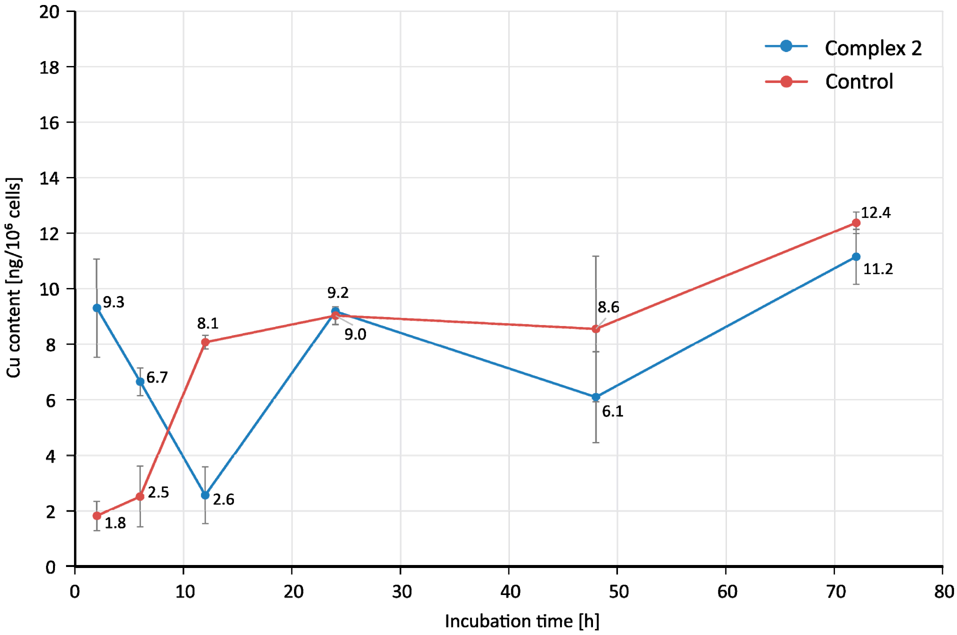

Figure S27. The time-dependent concentration profile of intracellular copper in the 22Rv1 cells treated by 3 µM solution of complex **2** after 24 h incubation and in untreated control.

Figure S28. The data regarding the interaction studies of complex **2** with selected proteins. **Panels A**, **B**, **C**.

Panel A. Molecular mass determination of model proteins by MALDI-TOF MS: MALDI-TOF mass spectra of intact proteins before and after the treatment with complex **2** were measured in linear positive ion mode using sinapinic acid as a matrix. No significant difference in the molecular mass was registered.

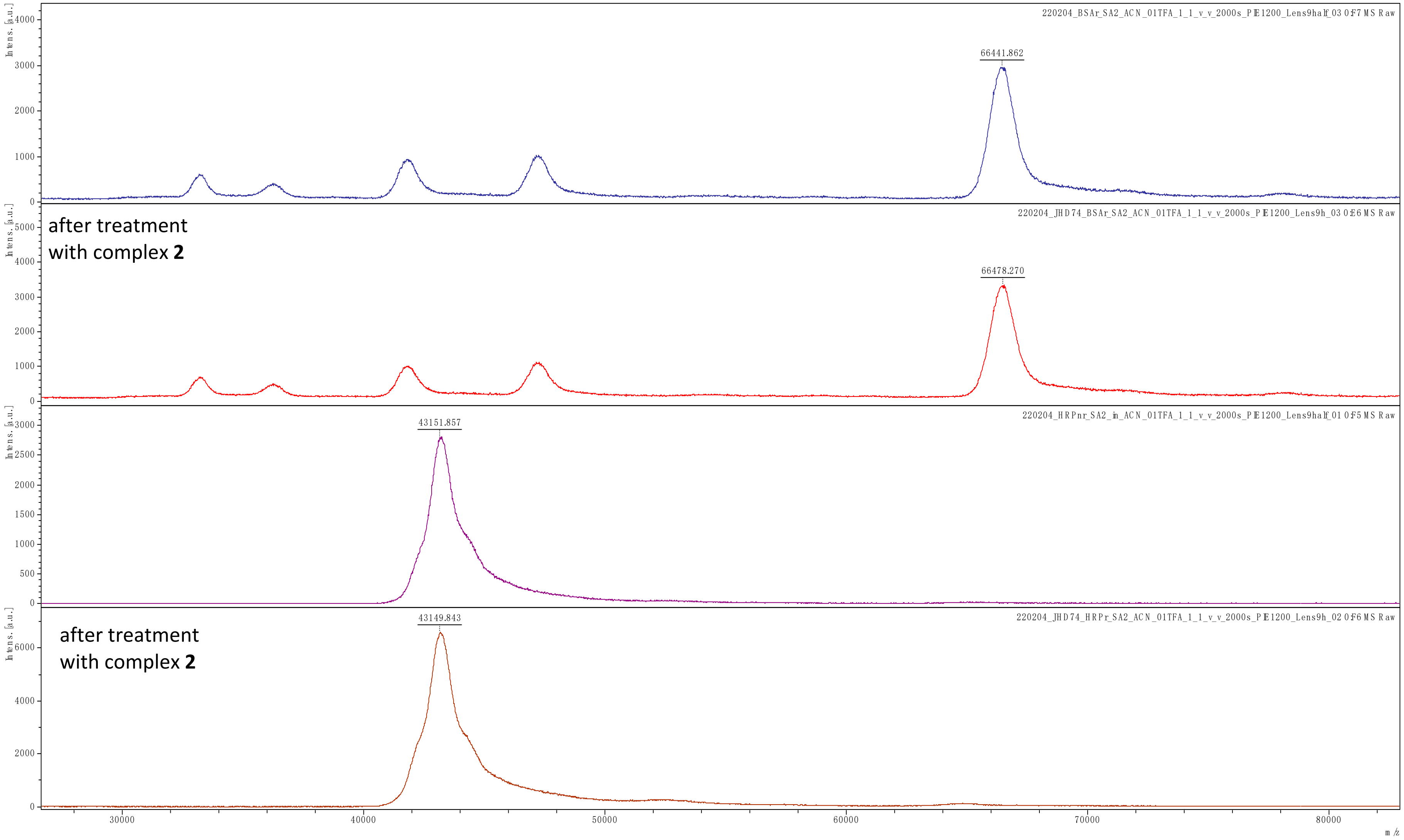

Panel B. Native polyacrylamide gel electrophoresis of model proteins: Bovine serum albumin (BSA) and rabbit glycogen phosphorylase B (GPB) were separated in a 12% polyacrylamide gel without or in the presence of 100 µM complex **2** inside the gel structure (pores). Protein amounts of 12.5 µg were loaded per sample well. The observed patterns show the existence of more protein forms (the separation under native conditions occurs based on the size and charge). It is evident that the mobility of the proteins was not influenced by the presence of complex **2**. The molecular mass of glycogen phosphorylase B is 200 kDa (a dimer under native conditions) and thus it partly did not migrate inside the 12% gel, which thus reduced the staining intensity compared to that of BSA applied in the same amount.

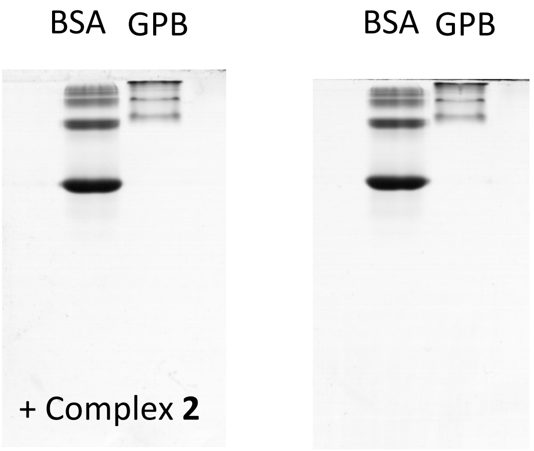

Panel C. Peptide mass fingerprinting of BSA and glycogen phosphorylase B: The stained protein bands after native electrophoresis (see in panel B) were excised from the gel slab and processed for in-gel protein digestion by trypsin. Peptides from the digests were purified and co-crystallized with alpha-cyano-4-hydroxycinnamic acid as a matrix on the target plate. MALDI-TOF mass spectrometry was then performed in the reflector positive ion mode. The panel shows mass spectra with BSA-derived peptides (top) and GPB-derived peptides (bottom). No important difference was found.

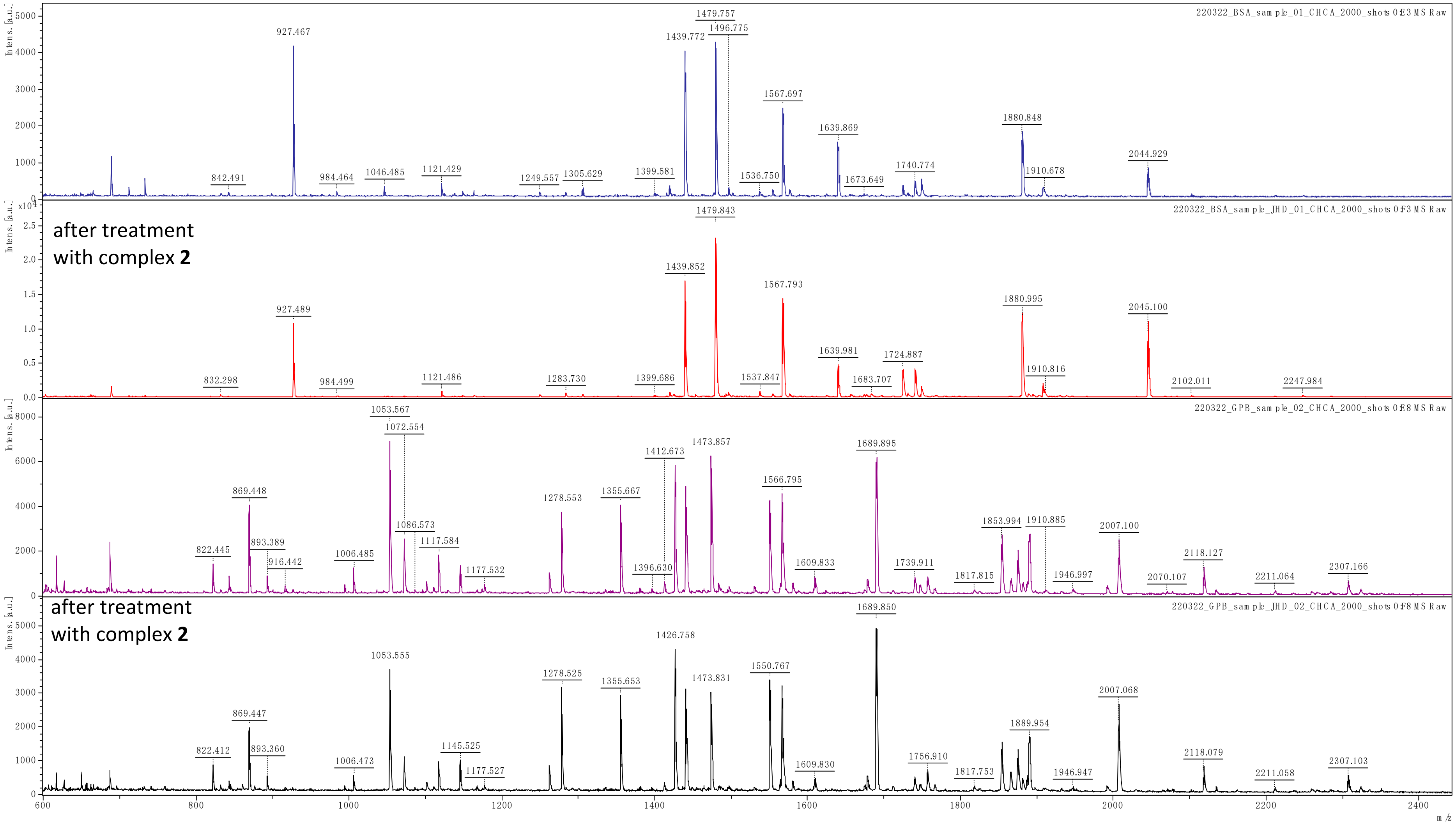

Supplement: Supplementary file 1 [file pharmaceutics-15-00307-s001.zip › pharmaceutics-2110986-supplementary.pdf]
